# Tissue-Resident Memory T Cells in Pancreatic Ductal Adenocarcinoma Coexpress PD-1 and TIGIT and Functional Inhibition Is Reversible by Dual Antibody Blockade

**DOI:** 10.1158/2326-6066.CIR-22-0121

**Published:** 2023-01-23

**Authors:** Hayden Pearce, Wayne Croft, Samantha M. Nicol, Sandra Margielewska-Davies, Richard Powell, Richard Cornall, Simon J. Davis, Francesca Marcon, Matthew R. Pugh, Éanna Fennell, Sarah Powell-Brett, Brinder S. Mahon, Rachel M. Brown, Gary Middleton, Keith Roberts, Paul Moss

**Affiliations:** 1 Institute of Immunology and Immunotherapy, College of Medical and Dental Sciences, University of Birmingham, Birmingham, United Kingdom.; 2 Centre for Computational Biology, University of Birmingham, Birmingham, United Kingdom.; 3 Nuffield Department of Medicine and Medical Research Council Human Immunology Unit, University of Oxford, Oxford, United Kingdom.; 4 Radcliffe Department of Medicine and Medical Research Council Human Immunology Unit, University of Oxford, Oxford, United Kingdom.; 5 Health Research Institute, Bernal Institute and School of Medicine, University of Limerick, Limerick, Ireland.; 6 University Hospitals Birmingham NHS Foundation Trust, Queen Elizabeth Hospital Birmingham, Birmingham, United Kingdom.

## Abstract

Pancreatic ductal adenocarcinoma (PDAC) has a poor clinical outlook. Responses to immune checkpoint blockade are suboptimal and a much more detailed understanding of the tumor immune microenvironment is needed if this situation is to be improved. Here, we characterized tumor-infiltrating T-cell populations in patients with PDAC using cytometry by time of flight (CyTOF) and single-cell RNA sequencing. T cells were the predominant immune cell subset observed within tumors. Over 30% of CD4^+^ T cells expressed a CCR6^+^CD161^+^ Th17 phenotype and 17% displayed an activated regulatory T-cell profile. Large populations of CD8^+^ tissue-resident memory (TRM) T cells were also present and expressed high levels of programmed cell death protein 1 (PD-1) and TIGIT. A population of putative tumor-reactive CD103^+^CD39^+^ T cells was also observed within the CD8^+^ tumor-infiltrating lymphocytes population. The expression of PD-1 ligands was limited largely to hemopoietic cells whilst TIGIT ligands were expressed widely within the tumor microenvironment. Programmed death-ligand 1 and CD155 were expressed within the T-cell area of ectopic lymphoid structures and colocalized with PD-1^+^TIGIT^+^ CD8^+^ T cells. Combinatorial anti–PD-1 and TIGIT blockade enhanced IFNγ secretion and proliferation of T cells in the presence of PD-1 and TIGIT ligands. As such, we showed that the PDAC microenvironment is characterized by the presence of substantial populations of TRM cells with an exhausted PD-1^+^TIGIT^+^ phenotype where dual checkpoint receptor blockade represents a promising avenue for future immunotherapy.

## Teaser

Immunotherapy has had little success in PDAC. The authors report an in-depth investigation of the PDAC tumor microenvironment that highlights PD-1 and TIGIT, or their corresponding ligands, as potential targets for combinatorial therapy to treat this devastating disease.

## Introduction

Pancreatic ductal adenocarcinoma (PDAC) is one of the greatest clinical challenges in oncology. The incidence of PDAC has increased in many countries and outcomes remain poor despite improvements in the delivery of chemotherapy regimens (1, 2). The dramatic advances associated with immunotherapy in many tumor settings have not been observed in the treatment of PDAC where checkpoint blockade is poorly effective (3). To develop more effective immunotherapy protocols for PDAC it is essential to increase understanding of the immune microenvironment within PDAC tumors and determine how this influences checkpoint expression on infiltrating T cells (4). Indeed, there is considerable interest in the mechanisms of immune evasion in the PDAC microenvironment (5). PDAC tumors are hypoxic and characterized by extreme desmoplastic reaction with intense fibroblastic proliferation. Despite this, tumor-infiltrating lymphocytes (TIL) are observed in many cases (6) and correlate positively with clinical outcome (7).

T cells within the tumor microenvironment often show features of functional exhaustion and this is typically associated with expression of programmed cell death protein 1 (PD-1), often in association with additional checkpoint markers such as Tim-3, LAG-3, or CTLA-4. Coexpression of checkpoint receptors has been reported in PDAC tumors and a heterogeneous profile has been observed between patients.

Here, we undertook a detailed characterization of the T-cell infiltrate in the tumor microenvironment of patients with PDAC and compared this with peripheral blood from the same patients. We observed large populations of CD4^+^ Th17 cells and regulatory T cells together with CD8^+^ tissue-resident memory (TRM) cells with very high levels of PD-1 and TIGIT coexpression. T-cell suppression by PD-1 and TIGIT engagement was reversible by dual checkpoint blockade. These findings reveal the complex profile of the T-cell infiltrate within PDAC and indicate dual PD-1 and TIGIT checkpoint blockade as a potential approach to ameliorate T-cell activity in this tumor of unmet need.

## Materials and Methods

### Participants

Forty-five patients with primary PDAC were recruited at University Hospitals Birmingham NHS Foundation Trust, Queen Elizabeth Hospital (Birmingham, United Kingdom) over a 4-year period (2016–2020). Peripheral blood and fresh tumor tissue was collected from each patient. The study design was approved by, and patient recruitment was carried out under, appropriate ethical approval by Birmingham Local Research Ethics Committee (REC 16/WM/0214). Written informed consent was obtained from patients, and studies were conducted in accordance with the Declaration of Helsinki. Inclusion criteria were as follows: male or female treatment-naïve patients undergoing pylorus-preserving pancreatico-duodenectomy after presentation with localized PDAC. The median age of participants was 66 years of age.

Formalin-fixed, paraffin-embedded (FFPE) PDAC tissue sections (*n* = 10 patients) were obtained from the Birmingham Human Biomaterials Resource Centre (HBRC; HTA Licence: 12358) ethically approved by North West – Haydock Research Ethics Committee (Ref 20/NW/0001; local ethics number 18–304). Slides were examined by an expert pathologist to determine the presence of pancreatic tumor ducts.

### Sample collection and processing

Peripheral blood mononuclear cell (PBMC) were isolated from heparinized blood by density gradient centrifugation within 2 hours of blood collection from the patient at the time of surgery. PDAC tumor tissue from the 45 patients was sampled by an expert pathologist within 1 hour of surgical resection, and enzymatically digested to a single-cell suspension using 1x Collagenase/Hyaluronidase (STEMCELL Technologies, 07919), 125 μg/mL Liberase-TL (Roche, 05 401 020 001) and 50 U/mL Benzonase (Sigma-Aldrich, E1014–25KU). The single-cell suspension was filtered through a 70-μm filter, and red blood cells were subsequently lysed. Cells were washed and resuspended in PBS (Sigma, D8537) or MACS Separation Buffer (Miltenyi Biotec, 130–091–221) prior to flow cytometric analysis, or cryopreserved in FCS (Sigma, F2442) containing 10% DMSO (Sigma, D2650) at a controlled rate of freezing in a Mr. Frosty, prior to cytometry by time of flight (CyTOF) analysis.

### Immunophenotyping

Multiparametric flow cytometry and CyTOF analysis was performed on matched PDAC patient PBMC and TIL. For flow cytometry, freshly isolated PBMC and TIL (1 × 10^6^) were resuspended in PBS containing human TruStain FcX Fc Receptor Blocking Solution (BioLegend, 422302) and Fixable Viability Dye eFluor 450 (Thermo Fisher, 65–0842–90) and incubated for 10 minutes at 4°C. Cells were then surface stained in MACS buffer with fluorophore-conjugated antibodies (Supplementary Table S1) for 20 minutes at 4°C. Data acquisition was carried out on a Gallios flow cytometer using CytoSoftware (Beckman-Coulter). Analysis was performed using FlowJo version 10.

For mass cytometry, previously cryopreserved PBMC and TIL (2×10^6^ to 4×10^6^ cells) were stained with metal-conjugated antibodies. Metal-conjugated antibodies were purchased from Fluidigm. Alternatively, unconjugated Maxpar-ready antibodies (BioLegend) were conjugated in-house with metal isotopes using the Maxpar X8 antibody labeling kit (Fluidigm). For cell staining, matched PBMCs and TILs were rested overnight and stained with 4 μmol/L Cell-ID Intercalator 103Rh (Fluidigm, 201103A) in GM media containing RPMI1640 (Thermo Fisher, 21875034), 10% FCS (Sigma, F2442), 1% penicillin/streptomycin (Thermo Fisher, 15070063) for 15 minutes at 37°C, for discrimination of dead cells. Cells were washed and resuspended in PBS containing human TruStain FcX Fc Receptor Blocking Solution (BioLegend, 422302) and incubated for 10 minutes at 4°C. Cells were then surface stained in MACS buffer with 2 separate cocktails of metal-conjugated antibodies focused on T cells (Supplementary Table S2) and myeloid cells (Supplementary Table S3) for 30 minutes at room temperature. Cells were washed in MACS buffer, then fixed with fresh 1.6% PFA in PBS for 10 minutes at room temperature. Cells were incubated with 0.125 μmol/L Cell-ID Intercalator-Ir (Fluidigm, 201192A) in Maxpar Fix and Perm Buffer (Fluidigm, 201067) overnight at 4°C. Prior to acquisition using the Helios instrument, cells were washed twice in MACS buffer and twice in MilliQ water. Cells were resuspended at 0.5 × 10^6^ cells/mL in 0.1X EQ beads (Fluidigm, 201078) in MilliQ water. Cells were acquired at a rate of 300 to 500 cells/s.

For mass cytometry data analysis, FCS files were processed and normalized using Helios CyTOF software (version 6.7; Fluidigm). FCS files were pre-gated manually using FlowJo software (version 10) to exclude EQ beads, cell doublets and nonviable cells. Further analysis was performed in FlowJo and Cytofkit2. In Cytofkit2, pregated FCS files (exported from FlowJo) were down sampled, ArcSinh transformed (cofactor = 5) followed by nonlinear dimensionality reduction and visualization by t-distributed stochastic neighbor embedding (t-SNE). Unbiased Louvain clustering (k = 30) using PhenoGraph was used to identify different clusters which were annotated on the basis of marker expression, where appropriate.

### Immunofluorescence staining

For immunofluorescence staining, FFPE PDAC tissue sections (*n* = 10) were dewaxed and dehydrated, followed by blocking of endogenous peroxidase activity in 0.3% H_2_O_2_. Antigen retrieval was performed using citrate buffer pH 6.0 at 100°C for 20 minutes, then slides were blocked with 2.5% normal horse serum (Vector Laboratories, S-2012–50) for 1 hour at room temperature. Primary antibodies (Supplementary Table S4) were applied, and slides were incubated overnight at 4°C. For visualization, Opal TSA fluorescent dyes were used (Akoya Bioscience, NEL810001KT) according to the manufacturer's protocol. Sections were counterstained with DAPI and imaged using a Zeiss Zen780 microscope with a 40x objective lens. Image analysis was performed using ImageJ (version 1.53t).

### Multiplex IHC (Lunaphore COMET platform)

Three representative PDAC cases underwent multiplex IHC on the Lunaphore COMET platform. FFPE PDAC tumor sections were dewaxed, and antigen retrieved on PT module (Thermo Fisher) in high pH buffer for 60 minutes at 102°C. Multiplex IHC was performed on the Lunaphore COMET using a sequential immunofluorescence protocol with an optimized panel of primary antibodies (Supplementary Table S5). Anti-mouse and anti-rabbit secondary antibodies were used at a concentration of 1:200 and 1:400 respectively. DAPI was used at 1:1,000. Before antibody staining cycles commenced, unstained images of the TRITC and Cy5 channels were captured for autofluorescence subtraction. Multilayer TIFF images were first exported to the Lunaphore viewer for initial quality control. Subsequent downstream analyses were performed in QuPath and R. On the basis of tumor annotations performed by an expert pathologist, the images were segmented into tumor and nontumor regions. Cellular segmentation was performed using CellPose (8).

### Checkpoint blockade assay

#### Generation of artificial antigen-presenting cell lines

A CHO cell–based artificial antigen-presenting cell system (CHO-aAPC) was used to conduct PD-1 and TIGIT blockade coculture experiments with T cells from PDAC patient PBMC. CHO cells expressing an anti-human CD3 (OKT3 clone) scFv and tetracycline-inducible programmed death-ligand 1 [PD-L1; a kind gift from Crescendo Biologics (Cambridge, United Kingdom)] were transduced to express human CD155 or CD112. Briefly, gBlocks (IDT) encoding the CDS region of CD155 or CD112 were cloned into the pRRLSIN.cPPT.PGK-GFP.WPRE Lentiviral vector (a gift from Didier Trono; Addgene plasmid #12252) in place of GFP and transfected into 293FT (Thermo Fisher, R70007; received May 2020) cells using Lipofectamine 3000 Transfection Reagent (Invitrogen) as per the manufacturer's instructions. Supernatant containing the virus was harvested after 48 hours and concentrated using a 100kDa Ultra 15-mL filter (Amicon). Concurrently, CHO-aAPC were seeded at 3×10^5^/well in a 12-well tissue culture plate (Greiner) in Hams F-12 media supplemented with 10% FCS, 1% penicillin/streptomycin and 1% GlutaMAX (Thermo Fisher, 35050061) and allowed to adhere overnight. The following day CHO-aAPC cells were transduced by spinfection with either CD155 or CD112 concentrated virus supplemented with 8 μg/mL Polybrene (Sigma H9268–5G). Cells were expanded for 72 hours, then harvested following Accutase (Thermo Fisher, A1110501) treatment for 10 minutes at room temperature. Cells were washed in MACS buffer and surface stained with anti-CD155-PE-Cy7 (SKII.4, BioLegend) or anti-CD112-PE (TX31, BioLegend) for 20 minutes at 4°C. Cells were subsequently FACS sorted (BD FACSMelody) on the basis of positive expression of CD155 or CD112 to a purity of > 98%. Stable expression of CD112 or CD155 on CHO-aAPCs was verified at regular intervals by flow cytometry. The resulting two cell lines allow constitutive expression of CD155 or CD112 in combination with inducible PD-L1. All cell lines were routinely tested for *Mycoplasma* contamination using the MycoAlert Mycoplasma Detection Kit (Lonza, LT07–418, last tested in June 2021). Cell lines were cultured up to a maximum of 25 passages. No additional authentication assays were performed.

#### CHO-aAPC: T-cell coculture assay

CD155-expressing CHO-aAPC cells, CD112-expressing CHO-aAPC cells, CHO-aAPC cells and parental CHO cells (negative controls) were irradiated (40 Gy) to arrest proliferation. Plates were seeded with each of the cell lines at 5×10^4^ cells/well of a 96-well flat-bottom tissue culture plate (Greiner) in 100 μL of Ham's F-12 media supplemented with 10% FCS, 1% penicillin/streptomycin, and 1% GlutaMAX. In wells where PD-L1 expression was required, 1-μg/mL doxycycline (Sigma, D5207–5G) was added. Plates were cultured at 37°C, 5% CO_2_ for 24 hours to allow cells to adhere and to induce PD-L1 surface expression. Following 24-hour incubation, doxycycline was removed, cells were gently washed and overlaid with 100-μL serum-free TexMACS media (Miltenyi Biotec, 130–097–196), then incubated for 1 hour at 37°C, 5% CO_2_.

During incubation, T cells were isolated from previously cryopreserved PDAC patient PBMC using the EasySep Human T Cell Isolation Kit (STEMCELL Technologies, 17951) as per the manufacturer's instructions. To monitor T-cell proliferation, isolated T cells were washed twice with PBS, and the cell pellet was resuspended in 1 mL of CellTrace Violet Dye solution (CTV, 1:1,000 in PBS, Thermo Fisher, C34571) per 1×10^6^ cells. Cells were incubated for 20 minutes at 37°C, then 10-fold TexMACS media was added and incubated for a further 5 minutes to quench remaining dye. T cells were counted, washed, and resuspending at 5×10^5^ cells/mL in fresh TexMACS media, and the required number of cells were aliquoted into 4 separate Eppendorf tubes. In each Eppendorf either 20 μg/mL of anti-TIGIT (mouse anti-human TIGIT, clone ID2, mutant D265A; a kind gift from Simon Davies, University of Oxford, United Kingdom), anti–PD-1 (Ultra-LEAF mouse anti-human PD-1, clone EH12.2H7; BioLegend), both anti-TIGIT and anti–PD-1 blocking antibodies, or isotype control antibodies were added. Cells were incubated for 30 minutes at room temperature to allow prebinding of antibodies to checkpoint receptors on T cells prior to coculture with CHO-aAPCs at 1:1 cell ratio. Coculture plates were centrifuged at 1,500 rpm for 5 minutes to mediate cell-to-cell contact between T cells and CHO-aAPCs before incubation at 37°C, 5% CO_2_.

The proliferative response of T cells harvested from CHO-aAPC:T cell coculture experiments was measured after 4 days by CTV dilution analysis using flow cytometry. Propidium iodide (PI; Sigma, P4864) was added prior to analysis to exclude nonviable cells. The quantitative detection and measurement of IFNγ and the effect of the anti-TIGIT and/or anti–PD-1 blockade on IFNγ production were measured by ELISA according to the manufacturer's instructions (Mabtech, 3420–1HP-1). Supernatants were removed from the assays plates following 4-day incubation and diluted 1 in 5 in TexMACS media. The ELISA was read using a Bio-Rad iMark plate reader at 450 nm and 655 nm.

### Droplet-based single-cell RNA sequencing

#### Sample preparation and FACS sorting

Single-cell suspensions of digested tumor tissues from 3 patients with PDAC were surface stained with anti-CD45 BV785 (2D1, BioLegend), anti-EpCAM APC (9C4, BioLegend), and anti-Podoplanin AF488 (NC-08, BioLegend) to confirm the presence of immune cells, epithelial cells, and fibroblasts, respectively. PI was added prior to sorting to exclude nonviable cells. Live CD45^+^ and CD45^–^ populations were sorted on a BD FACSMelody and adjusted to 1×10^3^ cells/μL.

#### Cell capture, library prep and sequencing

Samples with >85% cell viability were processed at the Genomics Birmingham Sequencing Facility (University of Birmingham, United Kingdom) for gene expression profiling using the 10X Genomics platform. Around 1.7×10^4^ cells per sample were processed using the Chromium Controller for a recovery of 1×10^4^ cells per sample, and library preparation was performed using the Chromium Single Cell 3′ Library & Gel Bead Kit v2 (CG00052, 10X Genomics) according to the manufacturer's instructions. Library quantification was performed using TapeStation (Agilent). Samples were sequenced on an Illumina NextSeq 500 (150 bps, paired-end) at a sequencing depth of >50,000 raw reads/cell.

Raw reads were processed using CellRanger (version 3, 10X Genomics) functions mkfastq and count. Raw bcl files were converted to fastq and aligned to the human reference genome GRCh38. Gene expression matrices for each patient were analyzed by R software (v3.6). Data preprocessing, quality control, dimensionality reduction, clustering and subsequent downstream analysis was performed using the Seurat package (v3.1.1; ref. 9). Sequencing data was retrieved on a total of 19,197 cells with medians of 3,535 unique molecular identifiers (UMI) and 1,197 genes per cell.

#### Preprocessing of sequence files

Using the Seurat package (v3.1.1; ref. 9), cells were filtered to keep only cells passing the following filters: (i) >500 UMIs, (ii) >200 and <3,500 genes, and (iii) <20% UMIs derived from mitochondrial RNA. Data was normalized, scaled, and variable features identified for each patient dataset using the Seurat function SCTransform.

#### Single-cell analysis to determine high-level cell type

Data from all patient samples were integrated using the Seurat SCTransform IntegrateData workflow using the top 3,000 variably expressed genes as the features for integration. Dimensionality reduction was applied using principal component analysis (PCA) on the 3,000 variably expressed genes and Uniform Manifold Approximation and Projection (UMAP) embedding determined using Principal Components (PC) 1:20. The selection of PCs was based on ElbowPlot and JackStrawPlot. A Shared Nearest Neighbor graph based on Euclidean distance in PCA space was constructed using the function FindNeighbours on PCs 1:20 and modules within this graph representing clusters were identified using the function FindClusters.

Clusters were annotated with their high-level cell type using a combination of automated cell type identification with cellassign (10) and canonical marker gene expression profiles. Differential gene expression between high-level cell types was performed by Wilcoxon Rank Sum Test using the function FindMarkers. Genes were regarded as positive marker genes for a given cell type if FDR < 0.01 and average logFC > 0.25 to all other high level cell types. Signature gene sets of CD8^+^ and CD4^+^ T cells taken from a pan-cancer study of immune cells in the microenvironment of solid tumors (11) were used to calculate CD4 and CD8 module scores of average expression using the Seurat function AddModuleScore.

#### Single-cell analysis of T cells to determine T-cell subpopulations

Data was subset to include only T cells, split by patient and stripped back to just the RNA count data then the same workflow as above (SCTransform, Integration and dimensionality reduction) was applied. Clustering was applied to the T cells using FindClusters with resolution of 1.8. Differential expression analysis between clusters was performed as previously. T-cell clusters were annotated with T-cell subtype using a combination of automated cell type annotation with SingleR (v1.0.6; ref. 12) on the immune cell reference sets from (13, 14) and expression of known subtype marker genes. Where cell type was not certain, the subset was annotated with the name of its top marker gene. Module scores were calculated for gene signatures defining regulatory T cells (11), exhaustion (*PDCD1, TIGIT, CTLA4, LAG3, LAYN, HAVCR2, CD244*) and TRM cells (15).

Average cluster expression profiles for genes of interest were calculated with the Seurat function AverageExpression and heatmaps visualized using the R package ComplexHeatmap (16).

### Survival analysis on The Cancer Genome Atlas data

For disease-free survival (DFS) analysis, GEPIA2 (17) was used to generate Kaplan–Meier curves of DFS and to calculate Cox proportional HR based on *ITGAE* gene expression from The Cancer Genome Atlas (TCGA)–pancreatic adenocarcinoma (PAAD) dataset database. This dataset consisted of RNA sequencing (RNA-seq) data from 178 PDAC cases and cases were grouped by low *ITGAE* expression (1st quartile) and high ITGAE expression (4th quartile) for comparison.

### Statistics

Statistical analysis was performed using GraphPad Prism version 8. Mann–Whitney test was used to determine differences between two independent groups. A Wilcoxon signed-rank test was used to compare nonparametric paired data. A *P* value of < 0.05 was considered statistically significant.

### Data availability statement

The single-cell RNA-seq (scRNA-seq) data has been deposited in the Gene Expression Omnibus under accession GSE210199. All other data are available in the main text or the Supplementary Materials or from the corresponding author on reasonable request, including the CyTOF data files.

## Results

### The PDAC immune microenvironment is enriched in **αβ** T cells that display a reduced CD4/CD8 ratio

**Figure 1.**
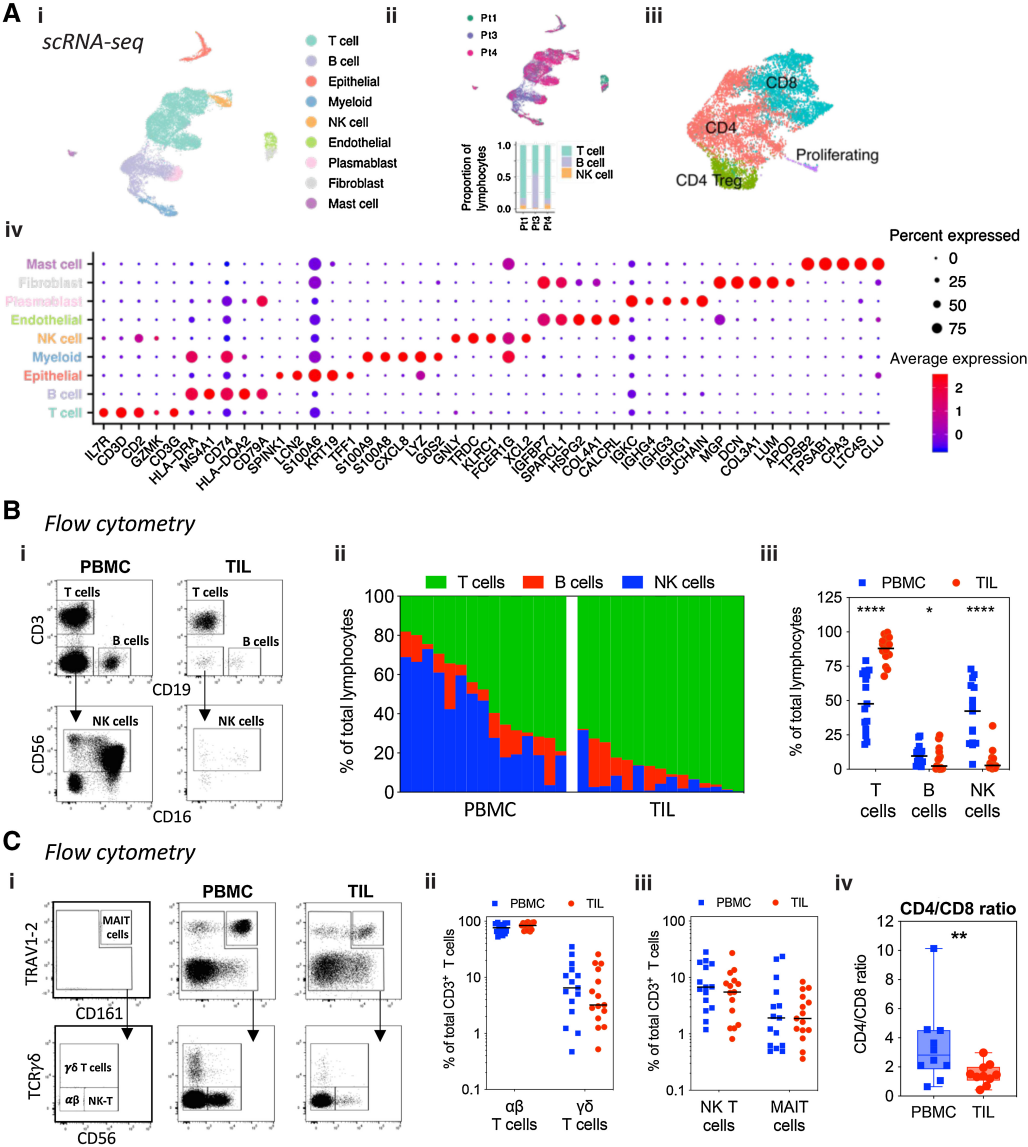
High-level cell type atlas of the PDAC tumor microenvironment. **A,** UMAP embedding of scRNA-seq data from 3 PDAC patient samples overlaid with high level cell type annotation (i). UMAP embedding overlaid with sample identification, and proportions of T, B, and NK cells identified in each sample (ii). UMAP embedding highlighting high level T-cell subsets (iii). Dotplot of the top markers expressed in each high-level cell type (iv). **B,** Representative plots showing T, B, and NK cell identification in matched PBMC and TIL from patients with PDAC by flow cytometry (i). Graphs showing proportions of T, B, and NK cells in PBMC and TIL (*n* = 15). Each bar in the waterfall plot represents a patient in (ii), and each dot represents a patient in (iii). **C,** Representative plots showing gating used to identify T-cell subsets in PBMC and TIL from patients with PDAC by flow cytometry (i). Quantification of αβ and γδ T cells (ii), and NK T and MAIT cells (iii) in PBMC and TIL (*n* = 15). Comparison of the CD4/CD8 T cell ratio between PBMC and TIL (*n* = 10) (iv). Horizontal lines represent median, boxes represent quartiles and whiskers represent min and max values. Data analyzed using Wilcoxon matched-pairs signed rank test. *, *P* < 0.05; **, *P* < 0.01; ****, *P* < 0.0001.

To analyze the immune repertoire of PDAC, we initially performed scRNA-seq on single-cell suspensions derived from tumor tissue of 3 PDAC cases. Core gene expression signatures were used to delineate nine predominant cellular subpopulations, and this defined that T cells represented the major lymphoid subset in PDAC (Fig. 1A; Supplementary Fig. S1). We then undertook flow cytometric analysis of T cells within peripheral blood and TIL isolated from surgically resected tumor tissue from patients who had undergone potentially curative pancreatico-duodenectomy.

T cells represented 48% of the peripheral lymphoid repertoire with an almost equivalent percentage of natural killer (NK) cells (42%) and a smaller proportion of B cells (Fig. 1B). In contrast, within the TIL population T cells comprised 88% of lymphocytes with a very low percentage of NK cells and B cells (Fig. 1B). In contrast to previous reports, 98% of T cells were αβ lineage and only 2% expressed a γδTCR (Fig. 1C). Nonconventional T-cell subsets including MAIT and NKT cells infiltrated PDAC tumors, but no differences were seen in the proportion of these cells between PBMC and TIL. A reduction of the CD4/CD8 ratio was observed within TIL populations, with a fall from 2.7 within blood to 1.1 within the tumor (Fig. 1C).

### CD4^+^ TIL contain few TRM and are highly enriched for T regulatory and th17-like populations

**Figure 2.**
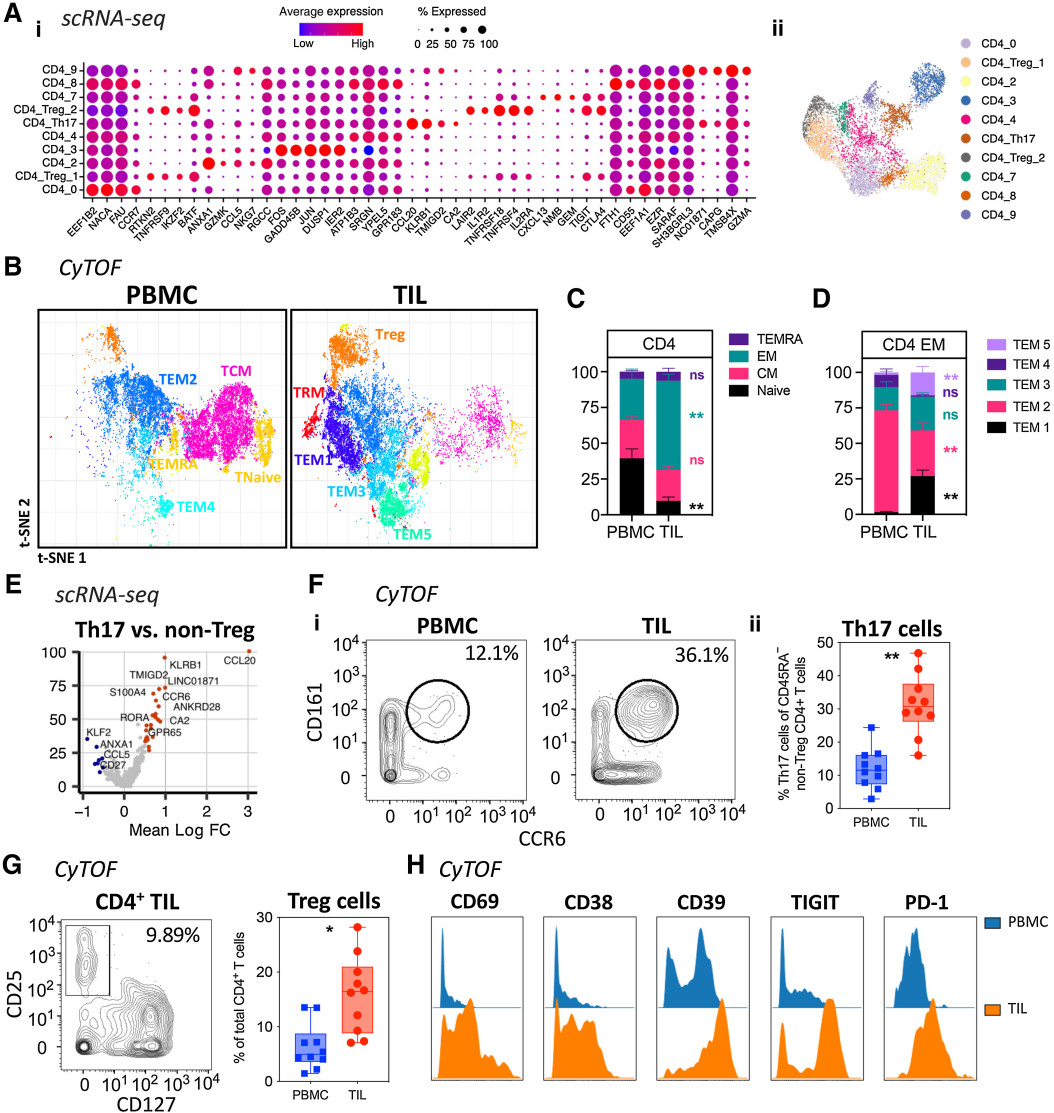
Characterization of CD4^+^ T-cell populations within the PDAC tumor microenvironment. **A,** Dot plot of the top markers expressed in each CD4^+^ T-cell cluster identified via Louvain clustering of scRNA-seq data from CD4^+^ T cells from 3 PDAC tumor tissue samples (i). Where identifiable in the data, clusters are annotated with known CD4^+^ T-cell phenotypes. UMAP embedding of CD4^+^ T cells overlaid with Louvain cluster labels (ii). **B,** A 35-parameter CyTOF analysis of CD45^+^ cells from PDAC patient PBMC and TIL (*n* = 10). t-SNE plots shows PhenoGraph-clustered CD4^+^ T-cell populations in PBMC and TIL. **C,** Stacked bar graph showing the proportion of Naïve, EM, CM, and TEMRA subsets in CD4^+^ T cells generated from the data in (**B**). **D,** Bar graph comparing the proportion of each annotated CD4^+^ EM subset (TEM1-5) in PBMC vs. TIL, generated using the data in (**B**). **E,** Differential expression analysis distinguishing CD4^+^ Th17 from other non-Treg CD4^+^ T-cells in scRNA-seq data, first presented in Fig. 1. Selected genes are labelled, and colored points indicate genes that are differentially expressed [BH adjusted *P* < 0.01 and absolute (average logFC) > 0.5]. **F,** Quantification of CD4^+^ Th17 based on dual expression of CCR6 and CD161, performed using the data in (**B**). Representative contour plots comparing Th17 in PBMC and TIL (i). Box and whisker plot comparing the proportion of Th17 among total memory (CD45RA^–^) non-Treg CD4^+^ T cells in PBMC and TIL (ii). **G,** Quantification of CD4^+^ Treg cells based on expression of CD25 and CD127, generated using the data in (**B**). Representative contour plot of Treg cells (CD25^+^CD127^low^) from PDAC TIL (i). Box and whisker plot comparing the proportion of Th17 cells in PBMC and TIL (ii). **H,** Histograms comparing expression levels of activation and differentiation markers on total Tregs from PBMC and TIL, generated using the data in (**B**). Horizontal lines represent median, boxes represent quartiles and whiskers represent minimum and maximum values. Data analyzed using Wilcoxon matched-pairs signed rank test. CyTOF comparisons analyzed using Wilcoxon matched-pairs signed rank test. *, *P* < 0.05; **, *P* < 0.01.

We next used scRNA-seq and CyTOF analysis to assess CD4^+^ T-cell heterogeneity within the tumor microenvironment. scRNA-seq identified 10 discrete CD4^+^ clusters, including two regulatory subsets, and cluster-defining genes included *ANXA1, CCL20, CCL20, TNFRSF4/18*, and *FTH1* (Fig. 2A; Supplementary Fig. S2). CyTOF analysis confirmed the predominant infiltration of T cells and the exclusion of NK-cell populations (Supplementary Figs. S3 and S4). Within the CD4^+^ T-cell subset, very few naïve cells (CD45RA^+^CCR7^+^) were found within TIL, but a marked expansion of effector populations was evident (Fig. 2B and C). The profile of effector memory populations was markedly different between PBMC and TIL with a decrease in the TEM2 subset (CD69^–^CD127^+^) and expansion of the TEM1 (CD69^+^CD127^+^) and TEM5 (CD57^+^PD-1^+^) subsets in TIL (Fig. 2D).

scRNA-seq analysis further highlighted a subset of CD4^+^ T cells expressing genes associated with Th17 differentiation, including *KLRB1, CCR6*, and *CCL20* (Fig. 2E). Then, CyTOF analysis confirmed the enrichment of a CD161^+^CCR6^+^ CD4^+^ T-cell population within TIL representing 31% of the effector pool compared with 12% in PBMC, indicating substantial expansion of Th17 cells within PDAC tumors (Fig. 2F).

Regulatory T-cell populations were also increased within PDAC, from 5% in PBMC to 16% within TIL (Fig. 2G). Furthermore, these CD4^+^ regulatory TILs were markedly more differentiated than cells within blood with high-level expression of CD69, CD38, CD39, TIGIT, and PD-1 (Fig. 2H).

### TRM CD8^+^ T cells reside in PDAC tumor tissue and correlate with improved clinical outcome

**Figure 3.**
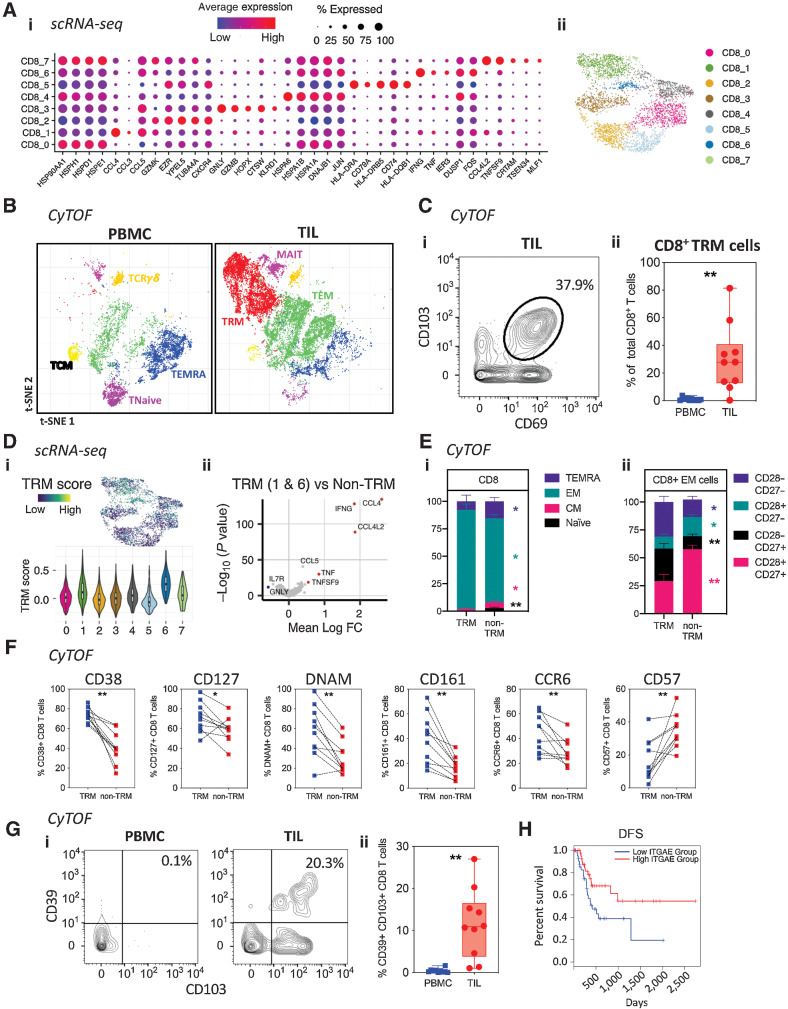
Characterization of CD8^+^ T-cell populations within the PDAC tumor microenvironment. **A,** Dot plot of the top markers expressed in each CD8^+^ T-cell cluster identified via Louvain clustering of scRNA-seq data (first presented in Fig. 1) from CD8^+^ T cells (i). UMAP embedding of CD8^+^ T cells from the 3 PDAC patient samples overlaid with Louvain cluster label (ii). **B,** CyTOF analysis of CD45^+^ cells from PDAC patient PBMC and TIL (*n* = 10), using data first used in Fig. 2B. t-SNE plots show PhenoGraph-clustered CD8^+^ T-cell populations in PBMC and TIL. **C,** Representative contour plot showing CD8^+^ TRM cells in PDAC TIL based on positive expression of CD69 and CD103, generated using the data in Fig. 2B (i). Box and whisker plot showing the proportion of CD8^+^ TRM cells in PBMC and TIL, generated using the data in Fig. 2B (ii). **D,** UMAP embedding, performed using scRNA-seq first presented in Fig. 1, overlaid with module score quintiles and module score distributions by CD8 T-cell cluster from scoring a core module of genes overexpressed in TRM T-cells (i). Differential expression analysis distinguishing TRM-like cells (clusters CD8_1 and CD8_6) from non-TRM cells (ii). Selected genes are labelled, and colored points indicate genes that are differentially expressed [BH adjusted *P* < 0.01 and absolute (average logFC) > 0.5]. **E,** Comparison of memory T-cell markers in CD8^+^ TRM and non-TRM in PDAC TIL, performed using the data in Fig. 2B. Bar graph comparing the proportion of Naïve, EM, CM, and TEMRA subsets in CD8^+^ TRM and non-TRM cells (i). Bar graph comparing the CD27 and CD28 expression pattern in CD8^+^ EM T-cells within TRM and non-TRM cells (ii). **F,** Line graphs comparing T-cell activation and differentiation marker expression on CD8^+^ TRM versus non-TRM cells in PDAC TIL, generated using the data in Fig. 2B. **G,** Representative contour plots (i) and quantification (ii) of CD39^+^ CD8^+^ TRM cells in PBMC and TIL, generated using the data in Fig. 2B. **H,** DFS analysis of patients with PDAC from the TCGA-PAAD dataset based on the expression level of *ITGAE* (CD103) in tumor tissue. Horizontal lines represent median, boxes represent quartiles and whiskers represent min and max values. Data analyzed using Wilcoxon matched-pairs signed rank test. *, *P* < 0.05; **, *P* < 0.01.

Given the increased presence of the CD8^+^ T-cell subset within PDAC TIL, we next assessed the profile of CD8^+^ T cells within the TIL population. scRNA-seq clustering analysis revealed 8 CD8^+^ subpopulations which were defined by expression of a range of genes including *CCL4, CXCR4, GNLY, HSPA6, HLA-DR, IFNG*, and *CCL4L2* (Fig. 3A; Supplementary Fig. S2).

CyTOF comparison of CD8^+^ T cells within blood and tumor identified expansion of effector cells within TIL with an associated decrease in naïve (CD45RA^+^CCR7^+^) and terminally differentiated effector memory (TEMRA; CD45RA^+^CCR7^–^) populations (Fig. 3B; Supplementary Fig. S5A). A further feature was the presence of large numbers of CD69^+^CD103^+^ TRM cells within TIL which represented an average of 28% of the CD8^+^ repertoire (Fig. 3C). A smaller population of CD69^+^CD103^+^ CD4^+^ T cells was also apparent within the tumor (Supplementary Fig. S5B).

Given the presence of CD8^+^ TRM within TIL we next used scRNA-seq and CyTOF to evaluate this population further. For TRM identification in the scRNA-seq dataset, each CD8 cluster was compared and scored against a core set of TRM genes (15) [Fig. 3D (i); Supplementary Fig. S6]. Differential gene expression profiling revealed that *IFNG, CCL4*, and *CCL4L2* were strongly upregulated within TRM clusters [Fig. 3D (ii)] whilst CyTOF showed that TRM were strongly enriched for the presence of effector memory (CD45RA^–^CCR7^–^) T cells with near complete exclusion of naïve and central memory (CD45RA^–^CCR7^+^) subsets [Fig. 3E (i)]. Furthermore, the profile of CD27 and CD28 expression on TRM populations showed them to be more highly differentiated than non-TRM effector subsets [Fig. 3E (ii)]. Increased expression of a range of effector and differentiation proteins was also seen on TRM including CD38, CD127, DNAM, CD161, and CCR6 (Fig. 3F). In contrast, CD57 expression was markedly reduced on TRM effector cells.

CD103 expression is a defining feature of TRM and the CD39^+^CD103^+^ phenotype is considered a potential marker of tumor-reactive T cells (18). Within PDAC TIL, CD39 was seen to be expressed on up to 27% (median: 11%) of the CD103^+^ CD8^+^ TIL population, potentially indicating a substantial tumor-specific T-cell pool (Fig. 3G).

Given the profound accumulation of CD8^+^ TRM effector cells in TIL from some patients with PDAC, we were interested to assess the potential clinical importance of this population. As such, GEPIA2 was used to perform survival analysis of the TCGA-PAAD tumor dataset based on the expression of *ITGAE*, the gene encoding CD103. The first and fourth quartiles represented ‘low’ or ‘high’ *ITGAE* expression, respectively. DFS was substantially increased with high levels of *ITGAE* (HR, 0.46; *P* = 0.029; Fig. 3H). Similarly, overall survival was also improved in patients with greater expression of *ITGAE* (Supplementary Fig. S7).

These data reveal that enrichment of highly differentiated CD8^+^ TRM T cells, which express IFNγ and CCL4, within the tumor microenvironment may play a role in patient survival.

### High levels of PD-1 and TIGIT coexpression are seen on CD4^+^ and CD8^+^ T cells in the PDAC microenvironment

**Figure 4.**
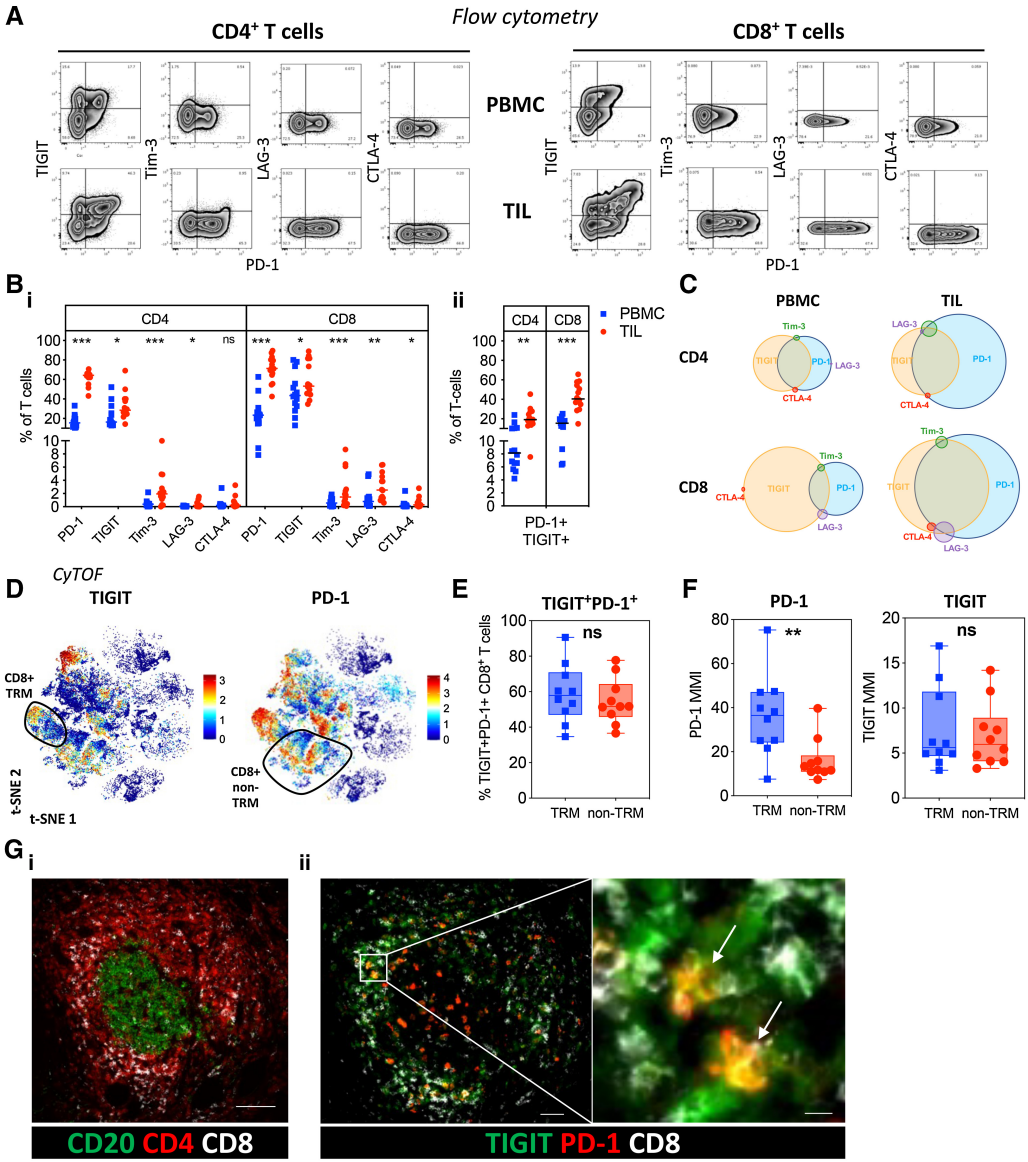
Checkpoint inhibitory receptor expression on PDAC T cells. **A,** Expression of checkpoint inhibitory receptors PD-1, TIGIT, Tim-3, LAG-3, and CTLA-4 on CD4^+^ and CD8^+^ T cells from matched PBMC and TIL was examined by flow cytometry (*n* = 14). Representative flow cytometric zebra plots show expression of each checkpoint inhibitory receptor alongside PD-1 expression for CD4^+^ and CD8^+^ T cells from PBMC and TIL. **B,** Scatter plots compare the proportion of each checkpoint receptor (i) and dual PD-1 and TIGIT expression (ii) on CD4^+^ and CD8^+^ T cells from PBMC and TIL. **C,** Venn diagrams show the overlapping expression of checkpoint inhibitory receptors on CD4^+^ and CD8^+^ T cells from PBMC and TIL. **D,** t-SNE plot of PDAC TIL CyTOF data first presented in Fig. 2B showing the expression level of TIGIT and PD-1. CD8^+^ TRM cells are highlighted. **E,** Box and whisker plot, generated using the CyTOF data first presented in Fig. 2B, compares dual TIGIT and PD-1 expression on TRM and non-TRM CD8^+^ T cells from PDAC TIL. **F,** Box and whisker plots, generated using the CyTOF data first presented in Fig. 2B, compare the MMI of PD-1 and TIGIT on TRM and non-TRM CD8^+^ T cells from PDAC TIL. **G,** Multiplex IHC staining shows T-cell staining around CD20^+^ B cells in lymphoid structures (scale bar: 100 μm; i); expression of PD-1 and TIGIT across the follicle with TIGIT focused within the T-cell zone (scale bar: 50 μm) and PD-1^+^TIGIT^+^ coexpression on CD8^+^ T cells (scale bar: 10 μm; ii). Horizontal lines represent median, boxes represent quartiles and whiskers represent minimum and maximum values. Data analyzed using Wilcoxon matched-pairs signed rank test. *, *P* < 0.05; **, *P* < 0.01; ***, *P* < 0.001.

Given the high proportion of effector T cells within TIL we next used flow cytometry to assess the pattern of coexpression of checkpoint proteins PD-1, TIGIT, Tim-3, LAG-3, and CTLA-4 on T cells within the tumor microenvironment and peripheral blood (Fig. 4A).

PD-1 was expressed highly within the tumor microenvironment and present on 64% and 71% of CD4^+^ and CD8^+^ T cells respectively within TIL. Increased expression of all the additional 4 checkpoint proteins was also seen on T cells within tumor. Tim-3, LAG-3, and CTLA-4 were present on a small proportion of cells whilst TIGIT was expressed on 28% and 53% of CD4^+^ and CD8^+^ T cells respectively [Fig. 4B (i)].

Given the high level of expression of PD-1 and TIGIT, we next assessed their pattern of coexpression. This showed large numbers of dual-positive PD-1^+^ and TIGIT^+^ T cells within TIL. These cells represented only 8% of CD4^+^ T cells within blood compared with 19% of CD4^+^ TIL. Comparable values for CD8^+^ subsets were 15% and 40% respectively [Fig. 4B (ii)].

Venn diagram representation of checkpoint expression, as determined by flow cytometry, confirmed the high level of coexpression of PD-1 and TIGIT and was then used to assess the relative distribution of the additional checkpoint proteins (Fig. 4C). Tim-3, LAG-3, and CTLA-4 were each expressed on a small proportion of cells, and these were nonoverlapping, suggesting a model whereby expression of each of these checkpoints is largely mutually exclusive.

The pattern of PD-1 and TIGIT expression was then mapped on to the t-SNE CyTOF analysis of CD8^+^ T cells within TIL (Fig. 4D). This showed relatively broad expression of both markers, in line with the flow cytometry data, and the proportion of dual-positive cells was similar in both the TRM and non-TRM subsets (Fig. 4E). The abundance of PD-1 was particularly focused on CD8^+^ TRM whereby the median metal intensity (MMI) of PD-1 expression was increased 3-fold on TRM populations compared with non-TRM cells (Fig. 4F). Although the relative expression of TIGIT was comparable between subsets, the MMI of TIGIT was significantly greater on CD39^+^ TRM compared with CD39^–^ TRM cells (Supplementary Fig. S8).

Next, multiplexed IHC staining was used to determine the anatomical location of PD-1^+^TIGIT^+^ CD8^+^ T cells. CD8^+^ T-cell populations were exclusively seen within the tumor stroma and at the periphery of the tumor and were not present within the glandular epithelium itself. Dual staining of PD-1 and TIGIT on CD8^+^ T cells was focused within dense aggregates of lymphocytes comprising CD4^+^ T cells surrounding B-cell clusters, in keeping with tertiary lymphoid structures (Fig. 4G).

These data reveal expression of a wide range of checkpoint proteins on T cells within PDAC TIL populations and a profound upregulation of PD-1 on PD-1^+^TIGIT^+^ CD8^+^ TRM.

### Ligands for PD-1 and TIGIT are expressed differentially on cells within the tumor microenvironment

Given the expansion of PD-1^+^TIGIT^+^ T cells on TIL, we next determined the expression of the ligands for PD-1 and TIGIT on cell populations within the tumor microenvironment. The ligands for PD-1 are PD-L1 and PD-L2 while TIGIT engages CD155 (PVR) and CD112 (PVRL2).

**Figure 5.**
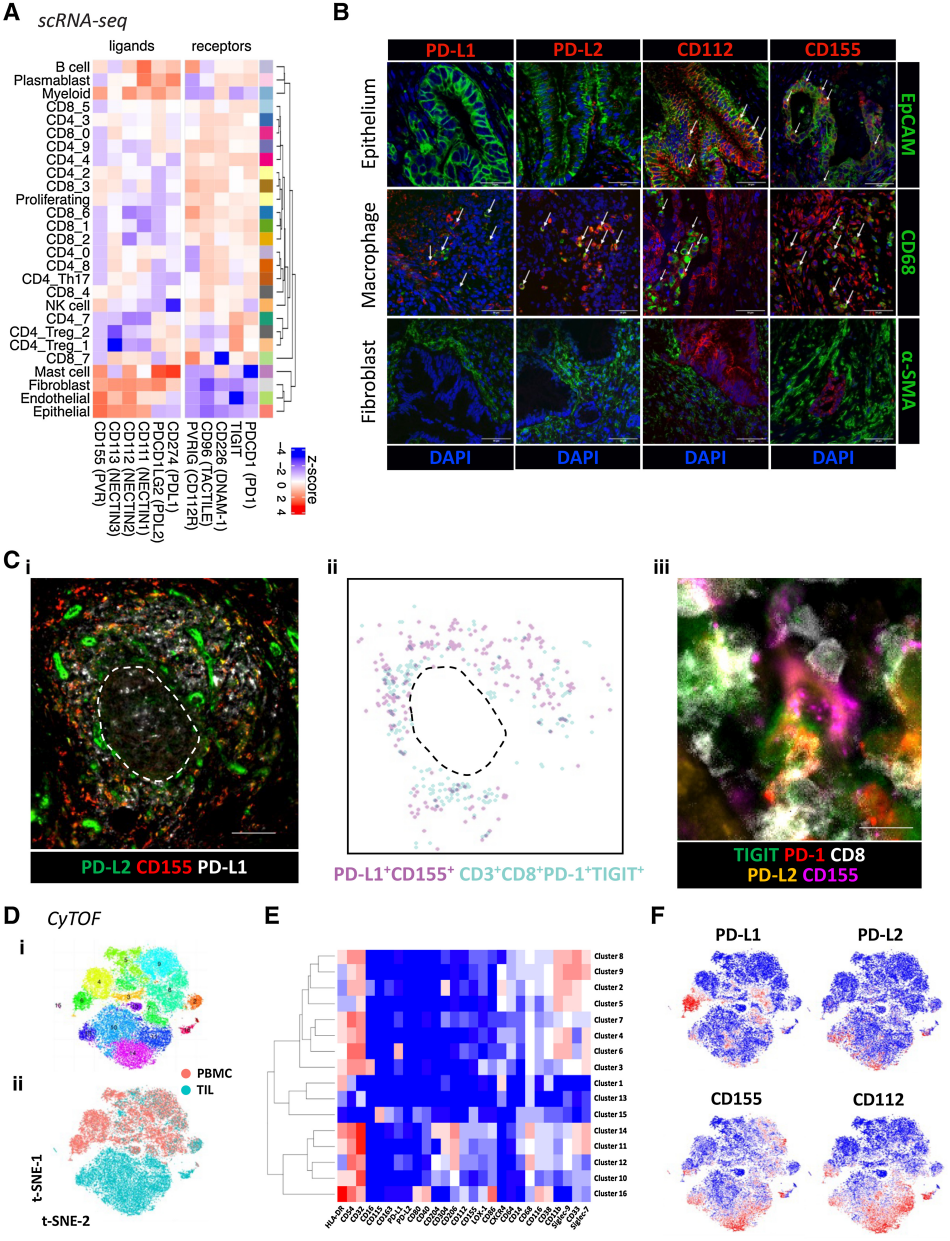
Expression of PD-1 and TIGIT ligands in the PDAC TME. **A,** Average expression profiles of TIGIT and PD-1 family receptors/ligands on all annotated cell subsets from scRNA-seq data first presented in Fig. 1. **B,** Representative confocal images of immunofluorescent staining for PD-1 and TIGIT ligands on tumor epithelium (EpCAM^+^), macrophages (CD68^+^), and stroma/fibroblasts (α-SMA^+^) using PDAC FFPE tissue (*n* = 10 patients). White arrows indicate examples of dual staining. Scale bars: 50 μm. **C,** Multiplex IHC staining of PD-1 and TIGIT ligands within ectopic lymphoid structures (scale bar: 100 μm; i). Digital representation of cell segmentation and localization of PD-L1^+^CD155^+^ cells and PD-1^+^TIGIT^+^ CD8^+^ T cells (ii). Direct engagement of a PD-1^+^TIGIT^+^ CD8^+^ T cell with a PD-L2^+^CD155^+^ cell within the T-cell area (scale bar: 10 μm; iii). Dashed line represents the border between the T- and B-cell areas. **D,** A 35-parameter CyTOF panel was used to determine the expression of PD-1 and/or TIGIT ligands on different myeloid-cell subsets in matched PBMC and TIL samples from patients with PDAC (*n* = 10). t-SNE plot shows PhenoGraph clusters of myeloid-enriched cell populations from combined PBMC and TIL (i), and cells stratified by sample type (ii). **E,** Heat map shows median expression level of key markers in each PhenoGraph cluster. **F,** t-SNE plots show the expression level of PD-1 and TIGIT ligands.

scRNA-seq confirmed PD-1 and TIGIT expression on T cells and demonstrated expression of additional TIGIT family members CD226, CD96, and CD112. PD-L1 and PD-L2 gene expression was seen within B-cell, myeloid-cell, and mast-cell subsets but not observed in tumor epithelial cells. In contrast, TIGIT ligands showed a differential and broader pattern of expression within endothelial and epithelial subsets (Fig. 5A).

These profiles were confirmed by immunofluorescence staining of FFPE PDAC tissues where PD-L1/L2 expression was seen predominantly on macrophage subsets whilst the TIGIT ligands CD112 and CD155 were also present on tumor cells (Fig. 5B; Supplementary Fig. S9). No expression of either PD-1 or TIGIT ligands was observed on α-SMA^+^ fibroblasts. Analysis using multiplex IHC further revealed that PD-L1/L2 and CD155 were expressed within the T-cell area of ectopic lymphoid structures, colocalizing with PD-1^+^TIGIT^+^ CD8^+^ T cells (Fig. 5C).

Next, CyTOF analysis of myeloid cells from PBMC and TIL was performed to assess the expression of PD-1 and TIGIT ligands on different myeloid-cell subsets (Fig. 5D). Within PDAC TIL, TIGIT ligands were broadly expressed across M2-like macrophage clusters 11 (HLA-DR^+^CD204^–^CD206^+^Siglec-7^+^), 12 (HLA-DR^–^CD204^+^CD206^+^Siglec-7^–^), and 14 (HLA-DR^+^CD204^+^CD206^+^Siglec-7^+^) whereas PD-L1 and PD-L2 expression was generally confined to cluster 12 (Fig. 5E and F; Supplementary Fig. S10). Expression of TIGIT ligands was generally absent on myeloid cells from PBMC.

These findings show that PD-1 and TIGIT on T cells can interact with ligands on a wide range of different cell subsets within the PDAC microenvironment.

### Blockade of PD-1 and TIGIT engagement partly rescues T-cell effector function

**Figure 6.**
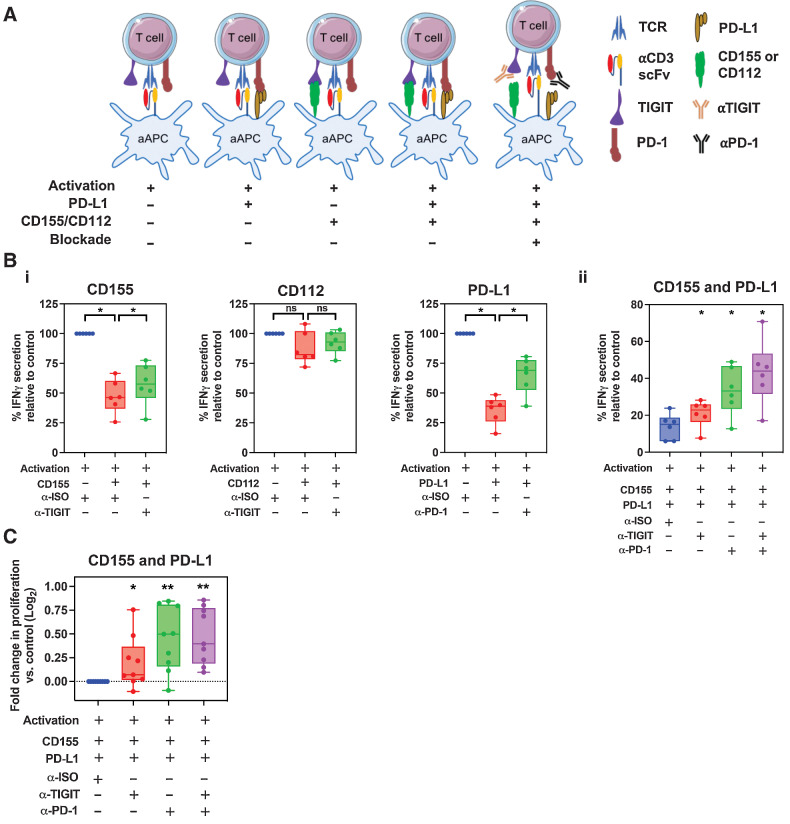
The effect of T-cell proliferation and cytokine secretion following anti–PD-1 and anti-TIGIT blockade. **A,** Schematic representation of the CHO-aAPC:T-cell coculture assay. T cells from patients with PDAC were cocultured with aAPCs expressing PD-1 and/or TIGIT ligands for 4 days in the presence of anti-TIGIT and/or anti–PD-1, or with a mAb isotype control. **B,** Cell culture supernatants from 6 patients in triplicate wells were harvested after 4 days of coculture and IFNγ was quantified by ELISA. Box and whisker graphs compare the levels of IFNγ secreted under 3 conditions—without ligand expression, with ligand expression, and for both ligand expression and mAb blockade with either CD155, CD112, or PD-L1 (i) or dual CD155 and PD-L1 (ii) expression. **C,** Proliferation of T cells (*n* = 9 patients) following coculture was determined by CTV dilution and analyzed by flow cytometry on day 4. Box and whisker graphs compare fold change in T-cell proliferation following single or dual CD155/PD-L1 mAb blockade compared with without (isotype mAb) blockade with CHO-aAPCs expressing both CD155 and PD-L1. Horizontal lines represent median, boxes represent quartiles and whiskers represent minimum and maximum values. Data analyzed using Wilcoxon matched-pairs signed rank test. *, *P* < 0.05; **, *P* < 0.01.

Given the very high frequency of PD-1 and TIGIT coexpression on T cells within the PDAC microenvironment, we were interested to determine how single or combined blockade of these receptors might act to modulate T-cell function. As such, T cells from PBMC of patients with PDAC were activated during coculture with a CHO-aAPCs expressing anti-human CD3 (OKT3) scFv. The aAPC was stably transduced to express either CD155 or CD112, and PD-L1 expression was controlled by a tetracycline inducible element. Cocultures of T cells with CHO-aAPCs were set up with differing combinations of PD-1/TIGIT ligand expression, with or without PD-1 and/or TIGIT blockade (Fig. 6A).

T-cell engagement with either CD155 or PD-L1, but not CD112, substantially suppressed IFNγ secretion after activation. Incubation with antibodies against TIGIT or PD-1 were able to partially reverse this suppression [Fig. 6B (i); Supplementary Fig. S11A]. Combinatorial engagement with CD155 and PD-L1 on target cells led to a profound 84% reduction in cytokine production. However, dual blockade with both TIGIT and PD-1 blocking antibodies led to a significant reversal of suppression with a 3-fold increase in IFNγ production [Fig. 6B (ii); Supplementary Fig. S11B].

We also investigated the influence of these interactions on the proliferative potential of T-cells. Again, antibody-mediated blockade of either TIGIT or PD-1 individually led to an increased proliferative response, although this was more pronounced with PD-1 blockade (Fig. 6C; Supplementary Fig. S12). No clear incremental effect on T-cell proliferation was observed following the addition of TIGIT blockade to PD-1 blockade.

These data show that engagement of PD-1 or TIGIT on T cells with their ligands can markedly reduce cytokine secretion and proliferation but these effects are ameliorated following blockade with PD-1 or TIGIT specific antibodies. Importantly, combinatorial antibody-mediated blockade of both PD-1 and TIGIT engagement acts to improve T-cell function and represents a promising therapeutic opportunity in this disease setting.

## Discussion

The failure of current immunotherapy regimens to transform the clinical outcome of patients with PDAC has led to increasing interest in understanding the mechanisms of immune evasion in this disease. Here, we examined the profile of checkpoint protein expression on T cells within the PDAC microenvironment and assessed how this may be overcome with antibody blockade.

T cells dominated the TIL population in PDAC, which is noteworthy given that T-cell infiltrate correlates with improved clinical outcomes (7). The great majority of these cells were of the αβ TCR lineage and in contrast to a previous report (19) only small populations of γδ T cells were present. T cells in TIL expressed an effector phenotype with a reduced CD4/8 ratio compared with blood, features that are also seen for the T-cell infiltrate in normal pancreas (20). No relative imbalance of NK T cells was seen within TIL despite the central role of this subset in regulating macrophage phenotype during development of murine PDAC (21). MAIT cells recognize conserved bacterial ligands and elevated numbers are seen in some tumor settings (22). However, proportions in PDAC TIL were stable even though bacterial colonization has been suggested to play a contributing role in the development of some PDAC tumors (23). In line with previous reports (24), the proportion of regulatory T cells was substantially increased within the PDAC microenvironment, representing 17% of the TIL infiltrate, and these cells were also seen to be highly activated, supporting continuing interest in the use of ipilimumab-based therapeutic combinations.

An unexpected finding was the presence of increased proportions of CCR6^+^CD161^+^ Th17 cells within PDAC. Increased numbers of IL21^+^ and IL26^+^ T cells have also been seen in PDAC and associate with an impaired clinical outcome, potentially through direct engagement with IL21R on tumor cells (25). There is increasing interest in the potential role of Th17 cells in tumor development and resistance to checkpoint inhibition and these findings indicate that consideration should also be given in relation to PDAC (26).

We observed large populations of TRM cells within the PDAC microenvironment, comprising 28% and 3.5% of the CD8^+^ and CD4^+^ repertoire, respectively. TRM comprise the largest proportion of cells in the body and play an important role in tissue homeostasis, including the potential for rapid functional response to antigen challenge (27, 28). CD8^+^ TRM have been identified as the predominant T-cell subset within the pancreas where they are focused within exocrine areas and regulated by local macrophage populations (20). Moreover, these populations have several features in common with tumor-associated TRM including a PD-1^+^CD57^–^IFNγ^+^ phenotype. The PDAC environment is markedly hypoxic and this, together with local TGFβ1 expression, may act to drive TRM production (29). Building on previous analysis of TRM within normal pancreas we were able to contrast TRM phenotype within PDAC. A proportion of TRM expressed an activated CD38^+^CD39^+^ phenotype within the tumor microenvironment. CD39^+^ expression has been correlated with tumor-specific recognition and is associated with reduced IFNγ and IL2 expression (30). These findings likely identify a substantial population of putative tumor-specific T cells within the CD39^+^ TRM compartment of PDAC tumors, like those described in other solid tumors (18, 31). Much less is known regarding the role of CD4^+^ TRM populations, although these have an important role in supporting development of tissue-resident B cells and CD8^+^ T-cell subsets (32), and can also mediate cytotoxic activity (33).

The TRM cells expressed a distinctive profile of checkpoint expression within the tumor. A high level of PD-1 expression was seen on the majority of CD4^+^ and CD8^+^ T cells but a marked increase in the expression of TIGIT was also apparent, in line with previous reports (34), and led to a substantial increase in the proportion of dual PD-1^+^TIGIT^+^ cells (35). Although this dual phenotype was present on both the TRM and non-TRM, the intensity of PD-1 was substantially increased on the TRM subset whilst TIGIT expression remained stable. In contrast, the proportion of cells that expressed Tim-3, LAG-3, or CTLA-4 was much lower, typically less than 5% of the TIL pool, although these were all expressed at increased levels compared with the peripheral T-cell pool. TIGIT expression appears to identify an exhausted CD8^+^ subset with high-level expression of *GZMK* and *EOMES* (34). Although it is also present on CD4^+^ regulatory T cells (36), less is known regarding its role on CD4^+^ effector cells (37). As such, a spectrum of checkpoint expression is observed within PDAC in which PD-1 is the dominant inhibitory molecule (38), closely followed by TIGIT and with only a minority of cells expressing additional proteins.

Given the high level of PD-1 and TIGIT expression on T cells within TIL, it became important to determine the profile of ligand expression on tumor, hemopoietic, and stromal cells. PD-1 ligand expression within PDAC correlates with impaired clinical outcome (7, 39–41), and PD-L1 was expressed mainly on hemopoietic subsets, such as B-cell, myeloid-cell, and mast-cell populations. Similar to previous reports, no expression of PD-L1 was seen on tumor cells (6, 42). Expression of TIGIT ligands CD112 and CD155 was seen on tumor cells and endothelial cells. Freed-Pastor and colleagues also showed that CD155 expression is widespread on the epithelium of murine PDAC and on 80% of human PDAC tumors, and may be driven by combined oncogenic *Kras* mutation and TP53 loss (43). In addition, we found abundant expression of CD112 and CD155 on immunosuppressive M2-like macrophages with CD204 and CD206 expression (44, 45). Myeloid cells have a key regulatory role in PDAC and are important targets for immunotherapy (46–48). It was noteworthy that CD8^+^ TRM populations were also seen to largely express DNAM-1 as this is a co-stimulatory ligand for CD155 and CD112 on T cells (49). However, the affinity of the CD155–TIGIT interaction is around 100-fold stronger than that between CD155–DNAM-1 (50) and is likely to underlie emerging evidence for the negative prognostic impact of combined TIGIT and CD155 expression in tumors (51). In addition, we identified cells expressing both PD-L1/L2 and CD155 within T-cell areas of ectopic lymphoid structures, a site of antigen presentation, which may have the potential to modulate antigen-specific T-cell responses.

Given the high level of coexpression of PD-1 and TIGIT on T cells, and their colocalization with PD-1/TIGIT ligand expressing cells within lymphoid follicles in PDAC tissue, it became of interest to assess the effects of dual checkpoint blockade on the function of T cells *in vitro*. Indeed, PD-1^+^TIGIT^+^ T cells are present in a range of other tumors (52–54) and are associated with poor outcome (55). Engagement with PD-L1 or TIGIT ligands at the time of T-cell activation suppressed cytokine production and proliferation and this was partially reversed by PD-1 blockade. A similar effect was also seen with TIGIT blockade but neither antibody alone was able to fully rescue the profile of cytokine secretion. Furthermore, differential responses were noted in relation to engagement with the TIGIT ligands CD155 or CD112, with stronger inhibition of cytokine production after binding to CD155, which suggests that this may represent a primary therapeutic target. Of note, PD-1 engagement had a more profound impact on T-cell proliferation than TIGIT binding. Combined engagement of both PD-1 and TIGIT led to marked functional impairment of T-cell function with ∼80% impairment of cytokine production. As such, it was noteworthy that dual PD-1 and TIGIT blockade acted in combination to overcome this, although this reversal was not complete and cytokine production remained suppressed by around 50%. CD155 can engage CD96 on T cells and impair antitumor responses (56, 57), so it is plausible that this interaction could be contributing to greater suppression, and reduced recovery, in our *in vitro* model. Combinatorial PD-1 and TIGIT blockade can elicit responses in murine models of PDAC (43) and these findings will help to guide optimal approaches to target TIGIT–CD115/CD112 interactions in cancer therapy (58). Neoantigen-specific T-cell responses have been demonstrated in patients with long term remissions from PDAC (59), but it is important to note that the antigen specificity of PD-1^+^TIGIT^+^ cells remain unknown (60). Nevertheless, dual PD-1 and TIGIT engagement within the tumor will substantially impair functional responses and that this may be partially overcome by checkpoint blockade.

In summary this analysis of the T-cell infiltrate within the PDAC microenvironment identifies expanded Th17 and CD4^+^ regulatory T subsets together with populations of PD-1^+^TIGIT^+^CD39^+^ CD8^+^ TRM cells. PD-1 and TIGIT ligands are expressed widely within the tumor bed but combined PD-1 and TIGIT checkpoint blockade can significantly overcome the immunosuppressive effect of PD-1 and TIGIT engagement on T-cell function. These findings provide new insights into the complex landscape of T cells within PDAC and should help to guide new approaches to immunotherapy.

## Supplementary Material

Supplementary Fig. S1 - S12 Supplementary Figures S1 - S12

Supplementary Tables S1 - S5 Supplementary Tables 1 - 5
