# Supplementary material for: Tissue-Resident Memory T Cells in Pancreatic Ductal Adenocarcinoma Coexpress PD-1 and TIGIT and Functional Inhibition Is Reversible by Dual Antibody Blockade
Source: Cancer Immunol Res. 2023 Jan 23;11(4):435–49. doi: 10.1158/2326-6066.CIR-22-0121 (PMC10068448; doi:10.1158/2326-6066.CIR-22-0121)
Supplement: Supplementary Fig. S1 - S12 — Supplementary Figures S1 - S12 [file cir-22-0121_supplementary_fig.s1_-_s12_suppsf1-sf12.pdf]

Supplementary Fig. S1

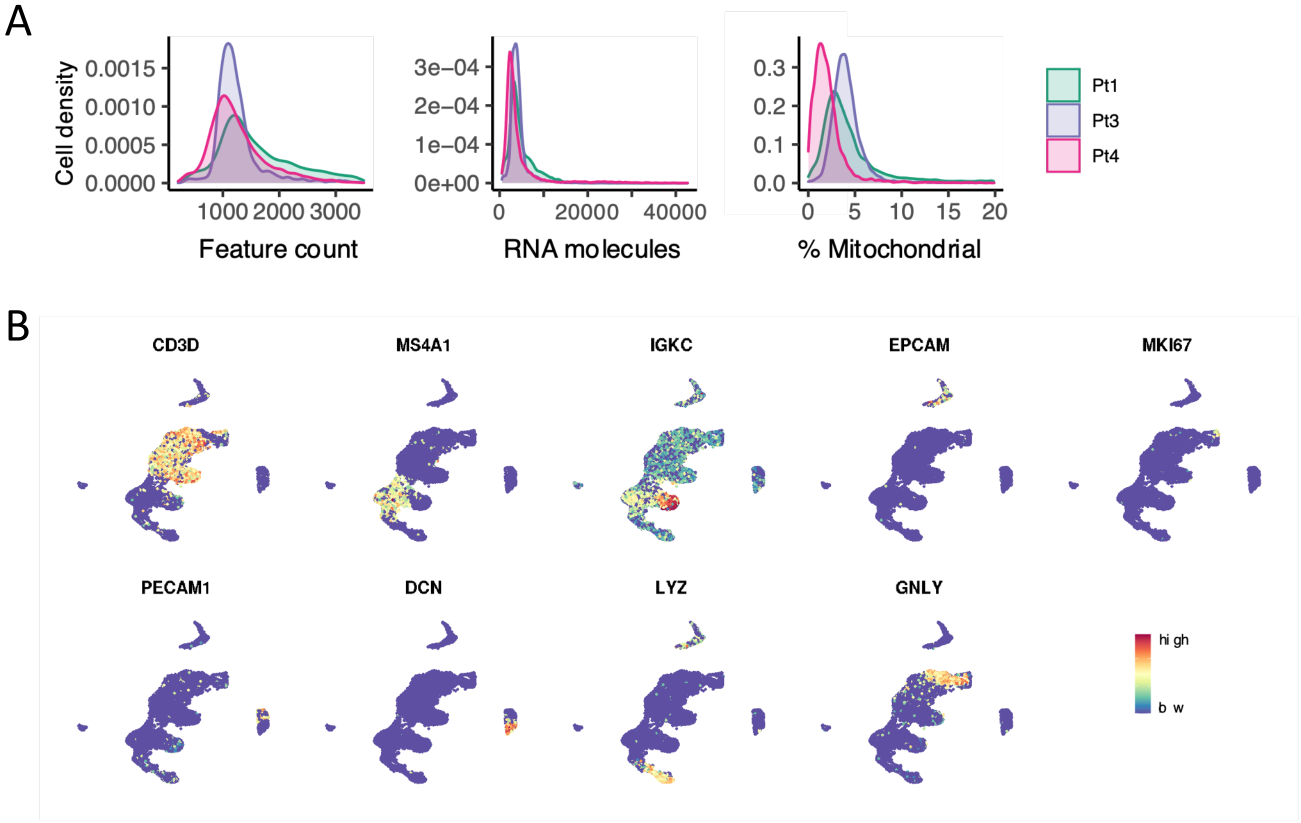

Fig. S1

A. Samplewise Distributions of scRNAseq feature counts, RNA molecule count and % reads mapping to mitochondrial genes

B. UMAP embedding of integrated scRNAseq data from PDAC samples Pt1, Pt3 and Pt4 overlaid with the raw count expression profile of canonical high level cell type marker genes

Supplementary Fig. S2

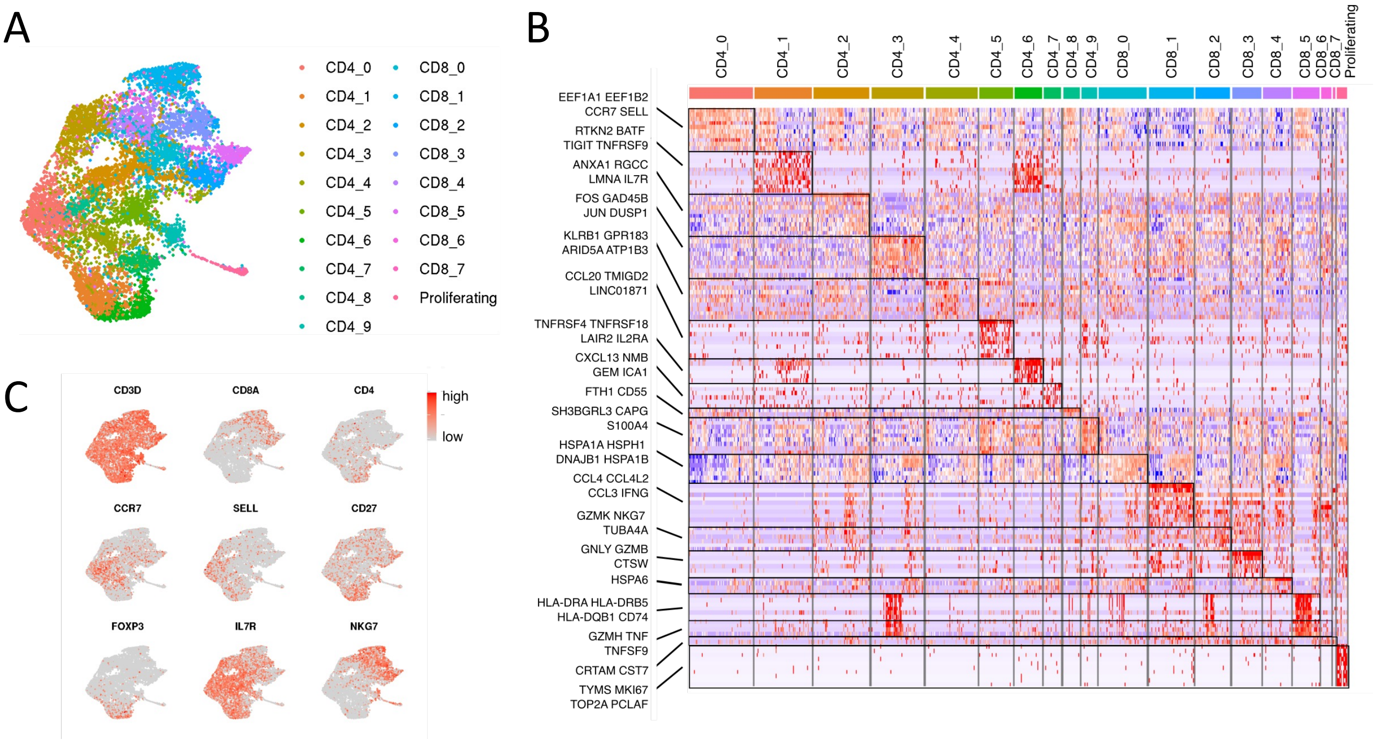

Fig. S2

A. UMAP embedding of T cell only scRNAseq data overlaid with cluster label identified by unsupervised clustering.
B. Normalized and scaled expression profile of the top marker genes identified as overexpressed in each cluster.
C. UMAP embedding of T cell data overlaid with raw count expression profile of canonical T-cell phenotypic markers of interest.

Supplementary Fig. S3

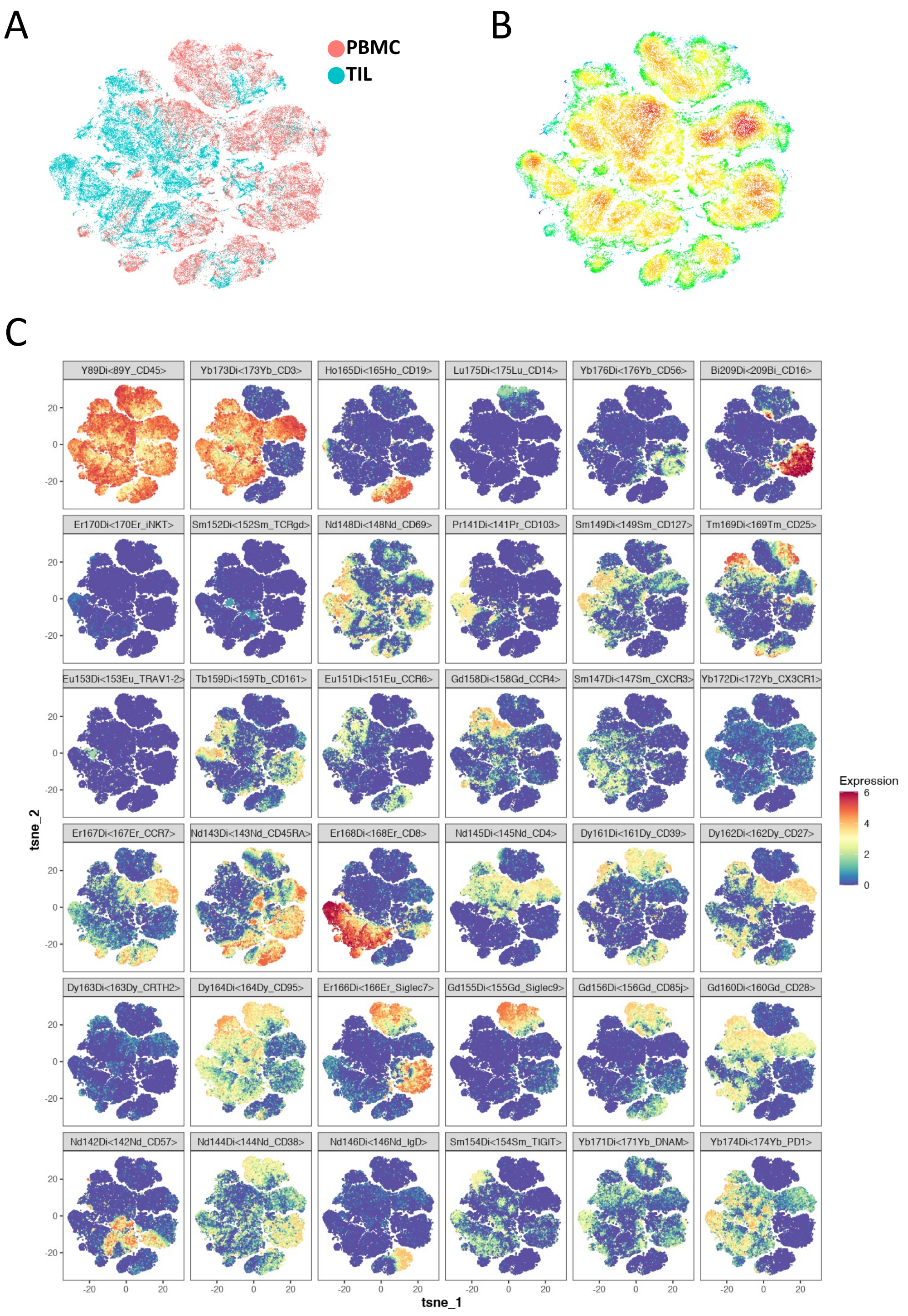

Fig. S3

A. t-SNE plots of combined PBMC and TIL CyTOF data coloured by sample type.

B. t-SNE plots of combined PBMC and TIL CyTOF data coloured by cell density.

C. Individual t-SNE plots of combined PBMC and TIL CyTOF data showing expression level of each marker of interest.

Supplementary Fig. S4

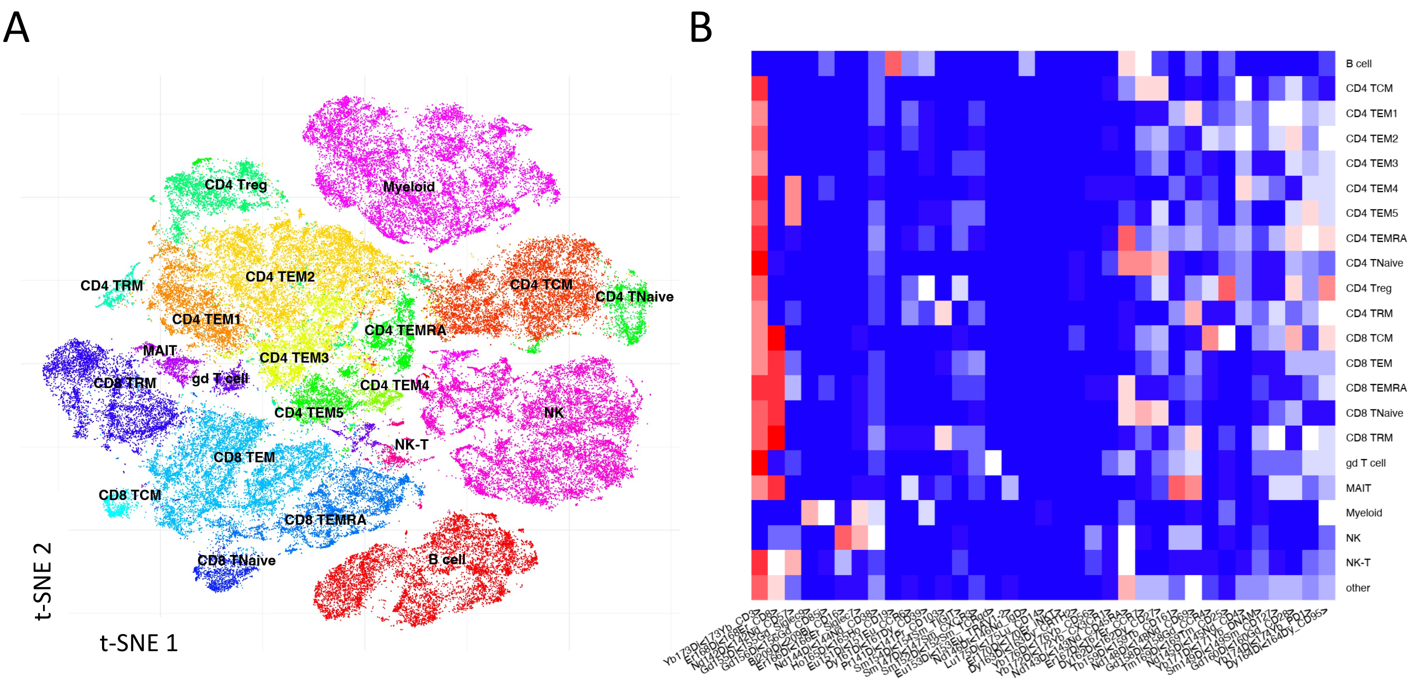

Fig. S4

A. t-SNE plot colored according to annotated Phenograph clusters of combined PBMC and TIL CyTOF data (n=10 patients).
B. Heatmap shows the expression profile of each marker within each annotated Phenograph cluster.

Supplementary Fig. S5

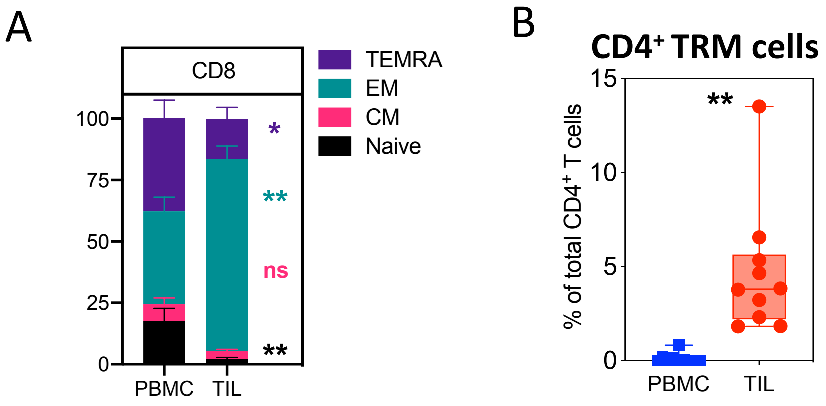

Fig. S5

A. Stacked bar graph showing the proportion of Naïve, EM, CM and TEMRA subsets in CD8+ T-cells from matched PBMC and TIL (n=10).
B. Box and whisker plot showing the proportion of CD4 TRM cells of total CD4 T cells in PBMC and TIL (n=10).

Comparisons analyzed using Wilcoxon matched-pairs signed rank test. *p<0.05, **p<0.01.

Supplementary Fig. S6

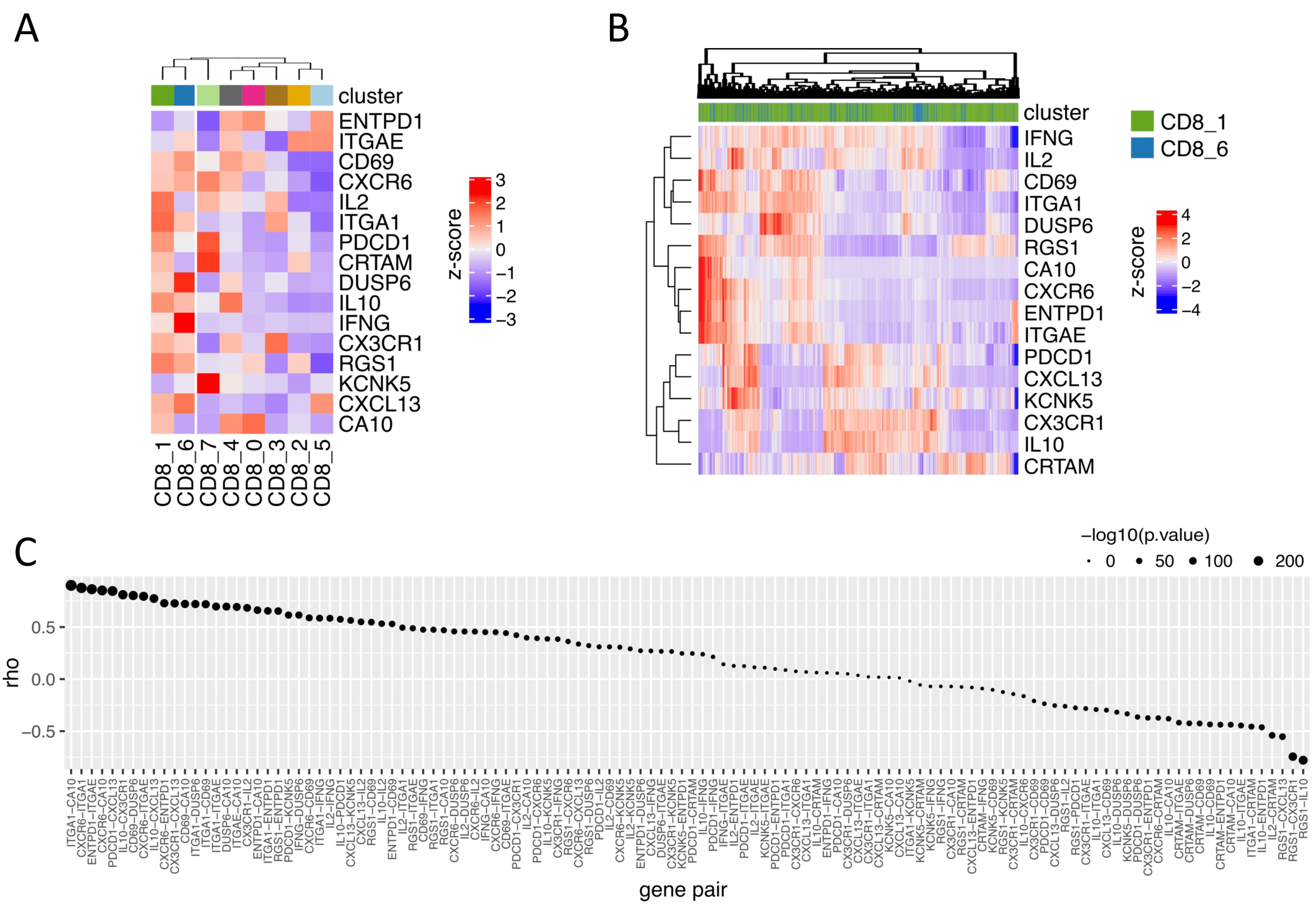

Fig. S6

A. Average within CD8 T cell cluster gene expression profile of a core set of TRM genes.

B. Single cell level imputed gene expression profile of a core set of TRM genes within TRM CD8 T cell clusters (CD8_1 and CD8_6).

C. Single cell level Spearmans pairwise correlations of TRM genes within cells from TRM clusters CD8_1 and CD8_6.

Supplementary Fig. S7

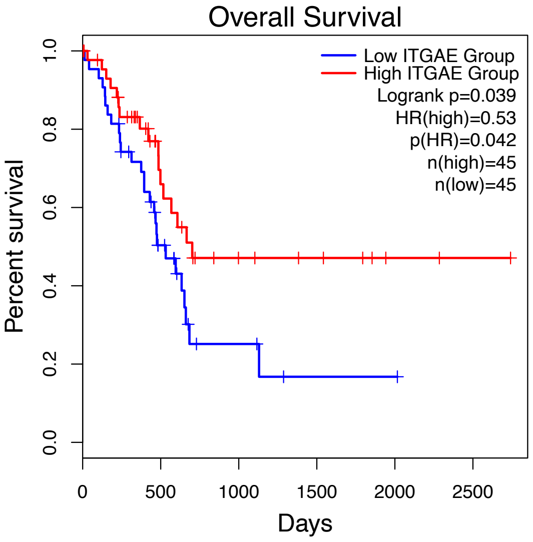

Fig. S7
Overall survival (OS) analysis of PDAC patients from the TCGA-PAAD dataset based on the expression level of ITGAE (CD103) in tumor tissue.

Supplementary Fig. S8

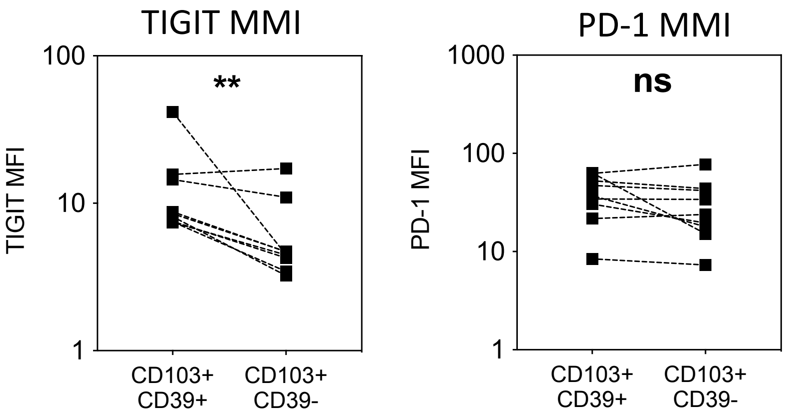

Fig. S8

Line graphs comparing the MMI of TIGIT and PD-1 on CD39+ and CD39- CD8+ TRM cells from PDAC TIL (n=10). Comparison analyzed using Wilcoxon matched-pairs signed rank test. **p<0.01.

Supplementary Fig. S9

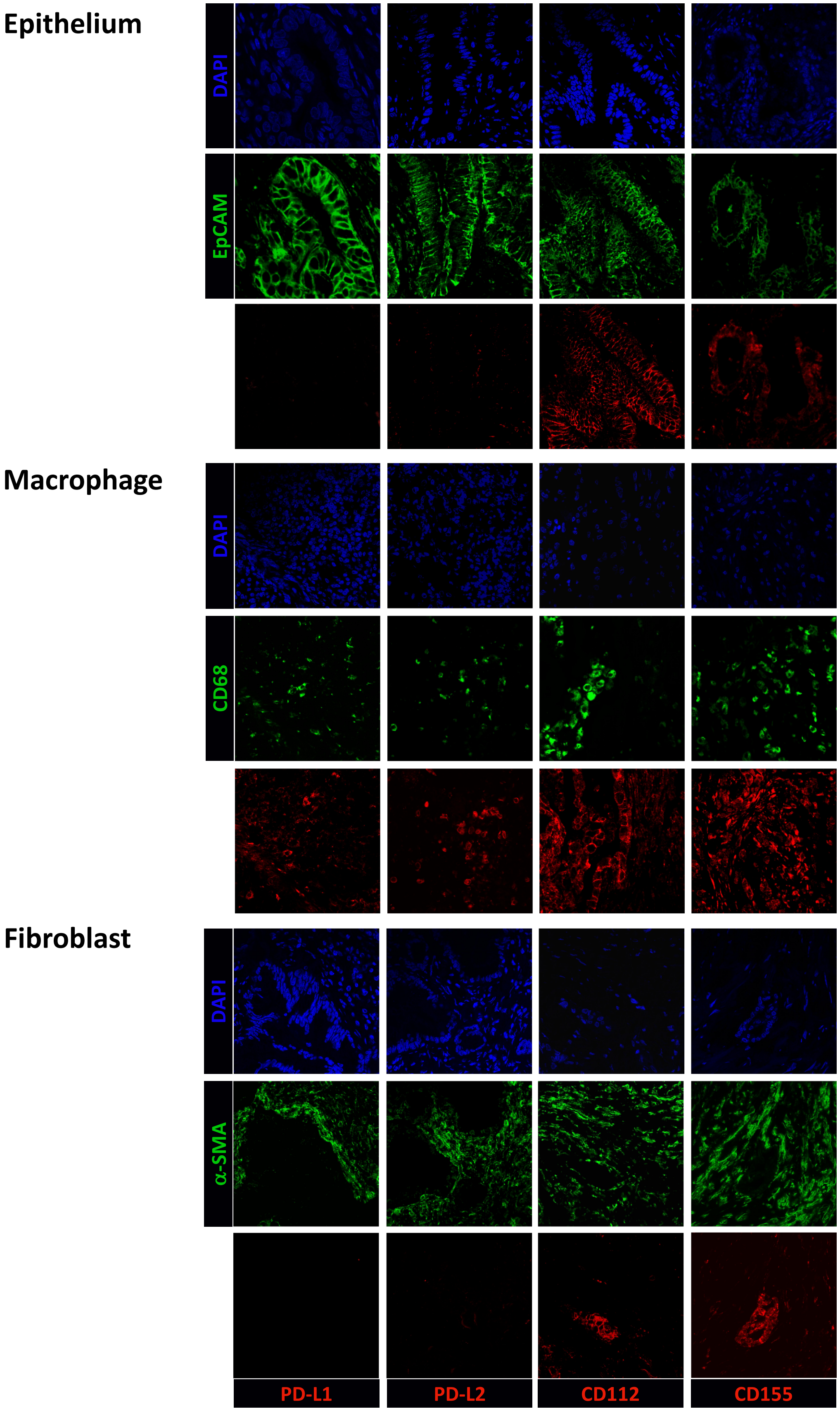

Fig. S9

Representative single color confocal images of immunofluorescent staining for PD-1 and TIGIT ligands on tumor epithelium (EpCAM+), macrophages (CD68+) and stroma/fibroblasts (α-SMA+) using PDAC FFPE tissue (n=10 patients).

Supplementary Fig. S10

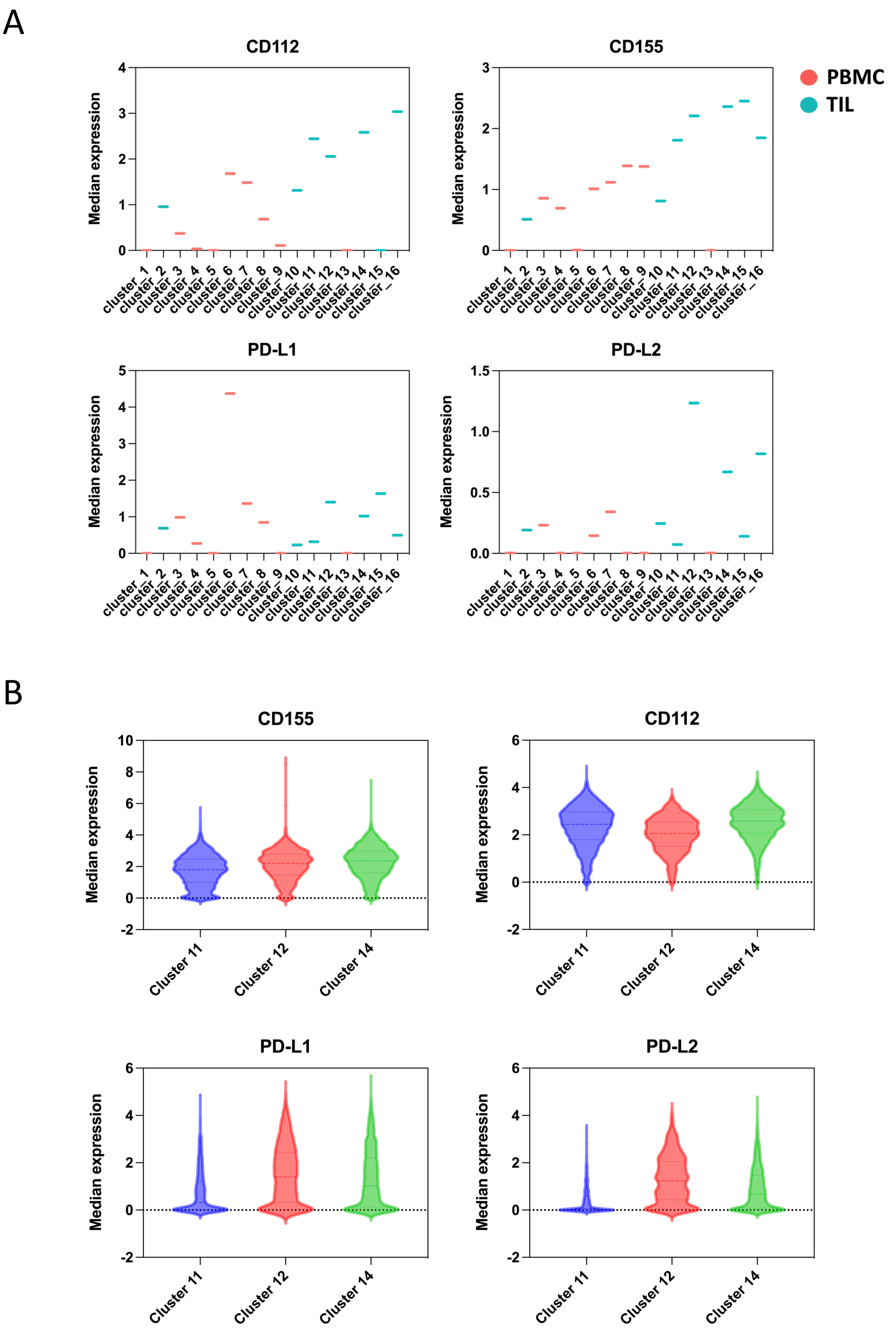

Fig. S10

A. Median expression of TIGIT and PD-1 ligands across all Phenograph cluster from myeloid-focused CyTOF panel (n=10 patients). Line colour represents the compartment (PBMC or TIL) of which the cluster is predominantly comprised.

B. Violin plots representing median expression on a per cell basis of TIGIT and PD-1 ligands across clusters 11 , 12 and 14.

Supplementary Fig. S11

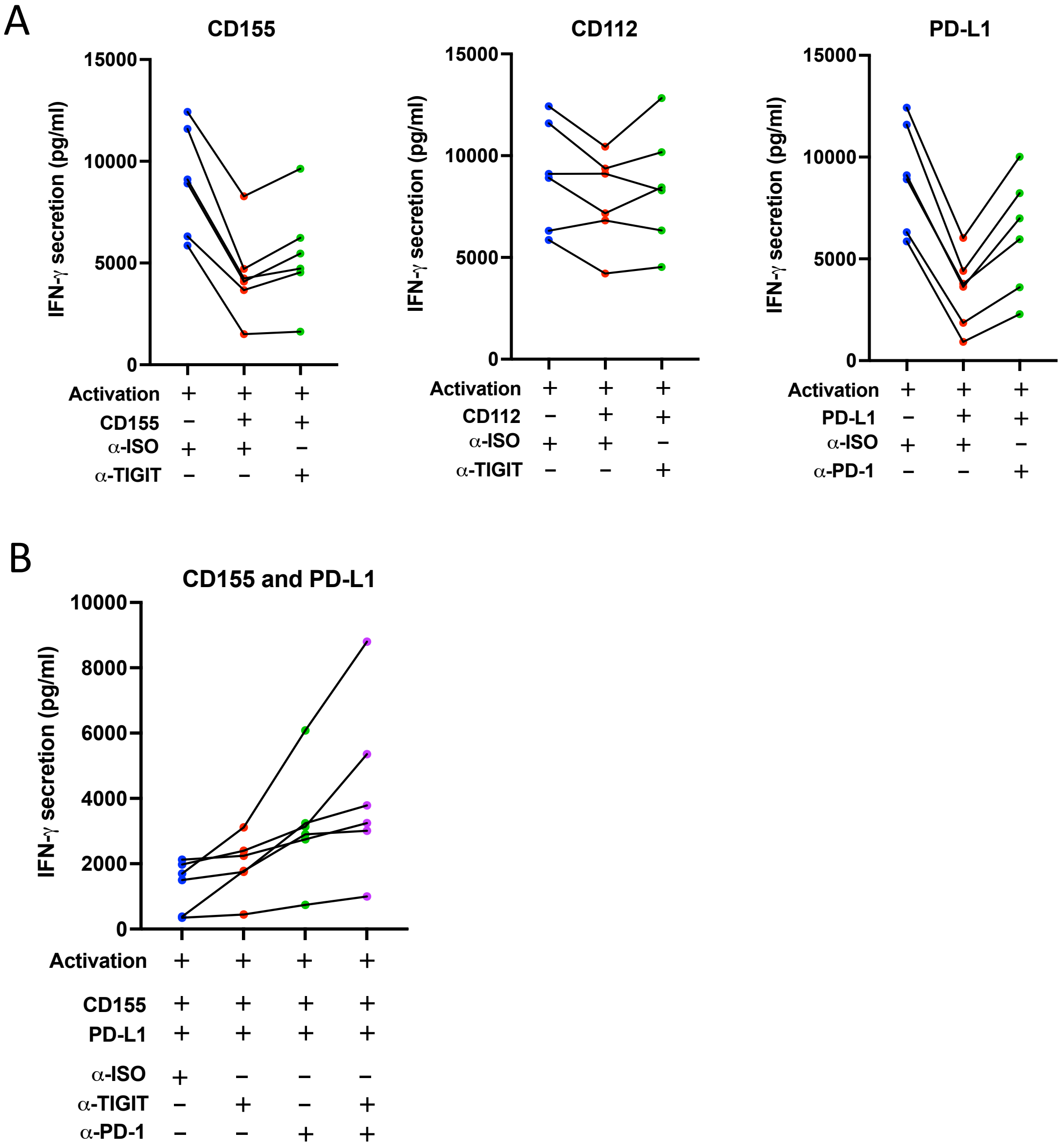

Fig. S11

IFN-γ production by T cells was quantified by ELISA following 4-day co-culture with aAPCs expressing A) CD155, CD112 or PD-L1, and B) dual CD155 and PD-L1. Monoclonal blocking antibodies pre-incubated with T cells are indicated on the x-axis. Each line represents an individual patient.

Supplementary Fig. S12

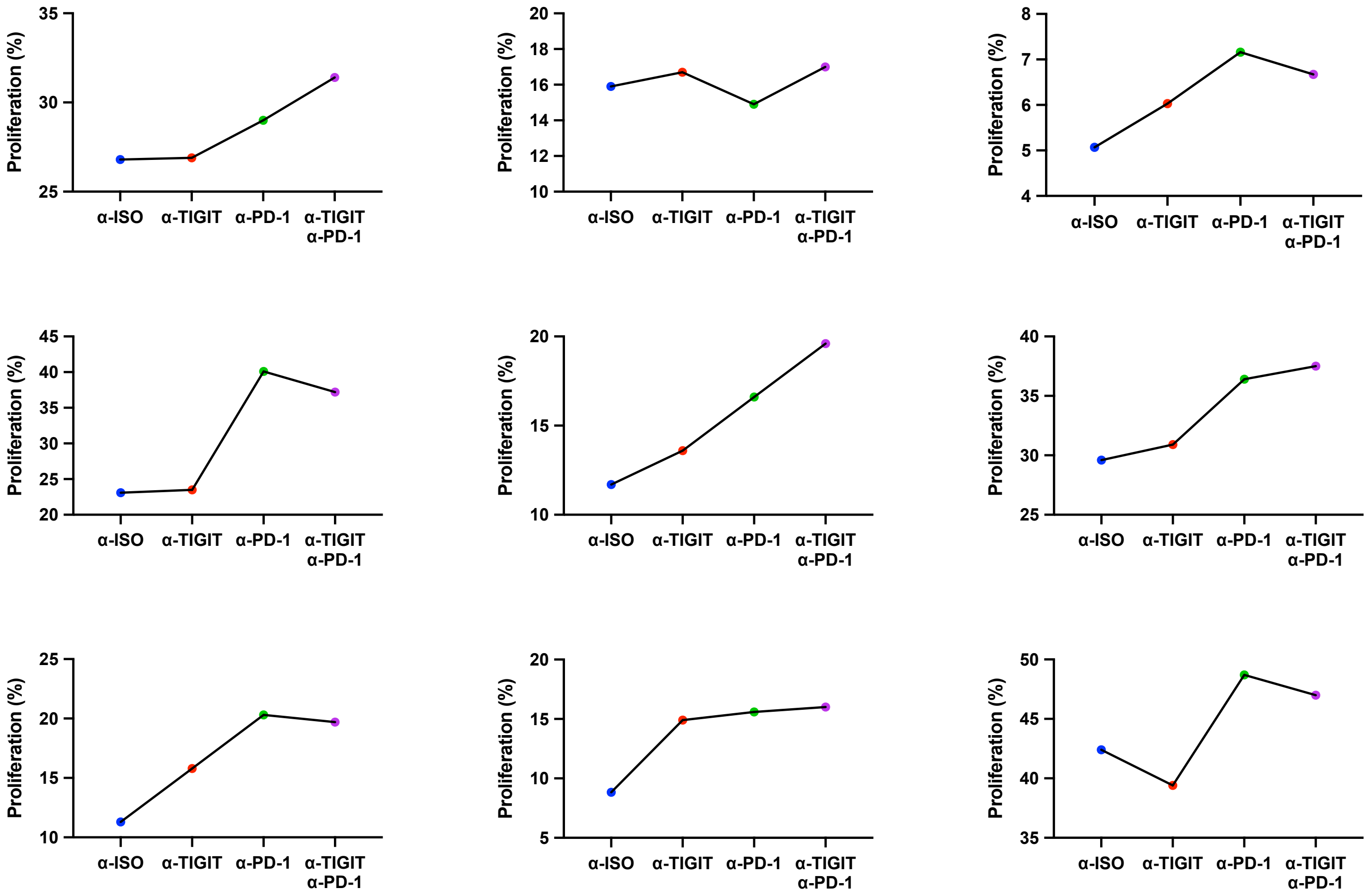

Fig. S12

Proliferation rate of T cells following 4-day co-culture with aAPCs expressing CD155 and PD-L1. For each condition, monoclonal blocking antibodies pre-incubated with T cells are indicated on the x-axis. Each line graph represents an individual patient.
