# Supplementary material for: Tissue-Resident Memory T Cells in Pancreatic Ductal Adenocarcinoma Coexpress PD-1 and TIGIT and Functional Inhibition Is Reversible by Dual Antibody Blockade
Source: Cancer Immunol Res. 2023 Jan 23;11(4):435–49. doi: 10.1158/2326-6066.CIR-22-0121 (PMC10068448; doi:10.1158/2326-6066.CIR-22-0121)
Supplement: Supplementary Tables S1 - S5 — Supplementary Tables 1 - 5 [file cir-22-0121_supplementary_tables_s1_-_s5_suppst1-st5.pdf]

Table S1. List of antibodies used for Flow Cytometry analysis

	Fluorophore	Marker	Clone	Supplier	Volume/test (µl)
**Checkpoint Panel**	ef506	Viability (Fixable)	65-0866-14	Thermo Fisher	0.1
	AF700	CD45	HI30	Biolegend	2.5
	FITC	CD3	UCHT1	Biolegend	5
	APC/Fire 750	CD4	RPA-T4	Biolegend	3.5
	PerCP/Cy5.5	CD8	SK1	Biolegend	3
	BV421	PD-1	EH12.2H7	Biolegend	4
	PE	TIM-3	F38-2E2	Biolegend	5
	PE-Cy7	LAG-3	11C3C65	Biolegend	5
	PE/Dazzle 594	CTLA-4	BNI3	Biolegend	5
	APC	TIGIT	MBSA43	Biolegend	5

	Fluorophore	Marker	Clone	Supplier	Volume/test (µl)
**Lymphocyte Quantification Panel**	ef506	Viability (Fixable)	65-0866-14	Thermo Fisher	0.1
	AF700	CD45	HI30	Biolegend	2.5
	FITC	CD3	UCHT1	Biolegend	5
	PE	TRAV1-2 (TCR Va7.2)	3C10	Biolegend	4
	PE/Dazzle 594	CD19	HIB19	Biolegend	5
	PerCP/Cy5.5	CD16	3G8	Biolegend	5
	PE-Cy7	CD56	HCD56	Biolegend	5
	APC	pan-$\gamma\delta$ TCR	B1	Biolegend	5
	APC/Fire 750	CD161	HP-3G10	Biolegend	5
	BV421	CD27	M-T271	Biolegend	4

Table S2. List of antibodies used for CyTOF analysis of T cells.

Metal conjugate	Marker	Clone	Supplier	Volume/test (µl)
**089Y**	CD45	HI30	Fluidigm	0.25
**103Rh**	Viability	201103A	Fluidigm	4 µM
**141Pr**	CD103	Ber-ACT8	Biolegend	1
**142Nd**	CD57	HCD57	Fluidigm	0.1
**143Nd**	CD45RA	HI100	Fluidigm	0.3
**144Nd**	CD38	HIT2	Fluidigm	1
**145Nd**	CD4	RPA-T4	Fluidigm	0.5
**146Nd**	IgD	IA62	Fluidigm	1
**147Sm**	CXCR3	G025H7	Fluidigm	1
**148Sm**	CD69	FN50	Fluidigm	1
**149Sm**	CD127	A019D5	Fluidigm	1
**150Nd**	HLA-DR	L243	Fluidigm	1
**151Eu**	CCR6	11A9	Fluidigm	1
**152Sm**	TCRγδ	11F2	Fluidigm	0.5
**153Eu**	TRAV1-2 (Va7.2)	3C10	Fluidigm	1
**154Sm**	TIGIT	MBSA43	Fluidigm	1
**155Gd**	Siglec 9	K8	Fluidigm	1
**156Gd**	CD85j	GHI/75	Fluidigm	1
**158Eu**	CCR4	L291H4	Fluidigm	1
**159Tb**	CD161	HP-3G10	Fluidigm	1
**160Gd**	CD28	CD28.2	Fluidigm	0.5
**161Dy**	CD39	A1	Fluidigm	0.5
**162Dy**	CD27	L128	Fluidigm	1
**163Dy**	CRTH2	BM16	Fluidigm	1
**164Dy**	CD95	DX2	Fluidigm	1
**165Ho**	CD19	HIB19	Fluidigm	0.25
**166Er**	Siglec 7	6434	Fluidigm	1
**167Er**	CCR7	G043H7	Fluidigm	1
**168Er**	CD8	SK1	Fluidigm	0.25
**169Tm**	CD25	2A3	Fluidigm	1
**170Er**	iNKT (Va24-Ja18)	6B11	Fluidigm	1
**171Yb**	DNAM	11A8	Fluidigm	1
**172Yb**	CX3CR1	2A91	Fluidigm	1
**173Yb**	CD3	UCHT1	Biolegend	0.25
**174Yb**	PD-1	EH12.2H7	Fluidigm	1
**175Lu**	CD14	M5E2	Fluidigm	0.5
**176Yb**	CD56	HCD56	Fluidigm	1
**191/193Ir**	Cell-ID Intercalator -Ir	201192A	Fluidigm	0.125 µM
**209Bi**	CD16	3G8	Fluidigm	0.5

Table S3. List of antibodies used for CyTOF analysis of myeloid cells.

Metal conjugate	Marker	Clone	Supplier	Volume/test (µl)
**089Y**	CD45	HI30	Fluidigm	0.25
**103Rh**	Viability	201103A	Fluidigm	4 µM
**141Pr**	IL-6R	UV4	Biolegend	1
**142Nd**	CD40	5C3	Fluidigm	1
**143Nd**	HLA-DR	L243	Biolegend	0.3
**144Nd**	CD38	HIT2	Fluidigm	1
**145Nd**	CD163	GHI/61	Fluidigm	1
**146Nd**	CD64	10.1	Fluidigm	0.5
**147Sm**	IFNGRa (CD119)	GIR-208	Biolegend	0.5
**148Sm**	PD-L1	29E.2A3	Fluidigm	1
**149Sm**	IL-10R	3F9	Biolegend	1
**150Nd**	CD86	IT2.2	Fluidigm	1
**151Eu**	IL-4Ra	G077F6	Biolegend	1
**152Sm**	CD112	TX31	Biolegend	1
**153Eu**	CD32	FUN-2	Fluidigm	1
**154Sm**	CD3	UCHT1	Fluidigm	1
**155Gd**	Siglec9	K8	Fluidigm	1
**156Gd**	CD204	7C9C20	Biolegend	0.75
**158Eu**	CD155	TX24	Biolegend	1
**159Tb**	Lox-1	15C4	Biolegend	1
**160Gd**	CD14	M5E2	Fluidigm	0.5
**161Dy**	CX3CR1	8F1/CXCR1	Biolegend	0.5
**162Dy**	CD80	2D10.4	Fluidigm	1
**163Dy**	CD33	WM53	Fluidigm	1
**164Dy**	CD116	4H1	Biolegend	0.5
**165Ho**	CD19	HIB19	Fluidigm	0.25
**166Er**	Siglec7	6434	Fluidigm	1
**167Er**	CD11b	ICRF44	Fluidigm	0.5
**168Er**	CD206	15-2	Fluidigm	1
**169Tm**	CD304	12C2	Fluidigm	1
**170Er**	CD54	HA58	Fluidigm	1
**171Yb**	CD68	Y1/82A	Fluidigm	0.25
**172Yb**	PD-L2	24F.10C12	Fluidigm	1
**173Yb**	CD115	9-4D2-1E4	Biolegend	1
**174Yb**	TIE-2	33.1 (Ab33)	Biolegend	1
**175Lu**	CXCR4	12G5	Fluidigm	1
**176Yb**	CD56	HCD56	Fluidigm	1
**191/193Ir**	Cell-ID Intercalator -Ir	201192A	Fluidigm	0.125 µM
**209Bi**	CD16	3G8	Fluidigm	0.5

Table S4. List of antibodies used for Immunofluorescence staining

Target	Clone	Isotype	Vendor
EpCAM	Polyclonal	Rabbit IgG	Abcam
CD68	KP1	Mouse IgG1	Dako Omnis
aSMA	1A4	Mouse IgG2A	R&D
CD155 (PVR)	Polyclonal	Rabbit IgG	Atlas Antibodies
CD112 (PVRL2)	Polyclonal	Rabbit IgG	Atlas Antibodies
PD-L1	IHC411	Rabbit IgG	GenomeMe
PD-L2	D7U8C	Rabbit IgG	Cell Signalling

Table S5. List of antibodies used for multiplex IHC on Lunaphore COMET platform

Mouse antibodies		
**Target**	**Clone**	**Vendor**
aSMA	1A4	Thermo Fisher Cat No. 14-9760-82
CD45	2B11+PD7/26	Agilent Cat no. M070101-2
CD8	4B11	Biorad Cat No. MCA1817
PanCK	AE1/AE3	Agilent Cat no. M351501-2
CD20	L26	CellMarque Cat No. 120M-86
anti-mouse IgG AF555	Polyclonal	Thermo Fisher Cat No A32727

Rabbit antibodies		
**Target**	**Clone**	**Vendor**
TIGIT	E5Y1W	Cell Signalling Cat No. 99567
PD-L2	D7U8C	Cell Signalling Cat No. 82723
PVR (CD155)	Polyclonal	Atlas Antibodies Cat No. HPA012568
PD-1	EPR4877(2)	Abcam Cat No. ab137132
PD-L1	IHC411	GenomeMe Cat No. IHC411-100
CD4	EPR6855	Abcam Cat No. ab133616
CD3	MRQ39	CellMarque Cat No. 103R-96
NA/K ATPase	EP1845Y	Abcam Cat No: ab76020
anti-rabbit IgG AF647	Polyclonal	Thermo Fisher Cat No. A32733
